# Comprehensive analysis of a novel signature incorporating lipid metabolism and immune-related genes for assessing prognosis and immune landscape in lung adenocarcinoma

**DOI:** 10.3389/fimmu.2022.950001

**Published:** 2022-08-25

**Authors:** Yuli Wang, Jing Xu, Yuan Fang, Jiefei Gu, Fanchen Zhao, Yu Tang, Rongzhong Xu, Bo Zhang, Jianchun Wu, Zhihong Fang, Yan Li

**Affiliations:** ^1^ Clinical Medical Center of Oncology, Shanghai Municipal Hospital of Traditional Chinese Medicine, Shanghai University of Traditional Chinese Medicine, Shanghai, China; ^2^ Information Center, Shanghai Municipal Hospital of Traditional Chinese Medicine, Shanghai University of Traditional Chinese Medicine, Shanghai, China; ^3^ School of Basic Medical Sciences, Fudan University, Shanghai, China

**Keywords:** bioinformatics, lung adenocarcinoma, lipid metabolism, immune, signature, prognosis, TCGA database, GEO database

## Abstract

**Background:**

As the crosstalk between metabolism and antitumor immunity continues to be unraveled, we aim to develop a prognostic gene signature that integrates lipid metabolism and immune features for patients with lung adenocarcinoma (LUAD).

**Methods:**

First, differentially expressed genes (DEGs) related to lipid metabolism in LUAD were detected, and subgroups of LUAD patients were identified *via* the unsupervised clustering method. Based on lipid metabolism and immune-related DEGs, variables were determined by the univariate Cox and LASSO regression, and a prognostic signature was established. The prognostic value of the signature was evaluated by the Kaplan–Meier method, time-dependent ROC, and univariate and multivariate analyses. Five independent GEO datasets were employed for external validation. Gene set enrichment analysis (GSEA), gene set variation analysis (GSVA), and immune infiltration analysis were performed to investigate the underlying mechanisms. The sensitivity to common chemotherapeutic drugs was estimated based on the GDSC database. Finally, we selected *PSMC1* involved in the signature, which has not been reported in LUAD, for further experimental validation.

**Results:**

LUAD patients with different lipid metabolism patterns exhibited significant differences in overall survival and immune infiltration levels. The prognostic signature incorporated 10 genes and stratified patients into high- and low-risk groups by median value splitting. The areas under the ROC curves were 0.69 (1-year), 0.72 (3-year), 0.74 (5-year), and 0.74 (10-year). The Kaplan–Meier survival analysis revealed a significantly poorer overall survival in the high-risk group in the TCGA cohort (*p* < 0.001). In addition, both univariate and multivariate Cox regression analyses indicated that the prognostic model was the individual factor affecting the overall survival of LUAD patients. Through GSEA and GSVA, we found that tumor progression and inflammatory and immune-related pathways were enriched in the high-risk group. Additionally, patients with high-risk scores showed higher sensitivity to chemotherapeutic drugs. The *in vitro* experiments further confirmed that *PSMC1* could promote the proliferation and migration of LUAD cells.

**Conclusions:**

We developed and validated a novel signature incorporating both lipid metabolism and immune-related genes for all-stage LUAD patients. This signature can be applied not only for survival prediction but also for guiding personalized chemotherapy and immunotherapy regimens.

## Introduction

As the leading cause of cancer-related mortality among all malignant tumors, lung cancer still contributes to a heavy burden not only for patients but also for all of society (1, 2). In China, the high prevalence of smoking and the risk of being exposed to second-hand smoke make the prevention and treatment of lung cancer grim (3). Lung adenocarcinoma (LUAD) is the dominant pathological category of non-small-cell lung cancer, and studies on the potential therapeutic targets of LUAD are constantly expanding. In addition to the common EGFR, ALK, and ROS1 inhibitors that are widely used, some small molecular drugs targeting rare driver mutations in LUAD such as BRAF, MET, RET, and NTRK, have also shown promise in clinical practice. However, resistance to targeted agents and distant metastasis remain the major causes of treatment failure. Therefore, there is an urgent need to seek potential therapeutic targets and prognostic markers to predict survival and guide the clinical treatment of LUAD patients.

In recent years, metabolic reprogramming has been regarded as one of the hallmarks of malignant tumors (4). Lipids are a critical form of energy storage in the human body, and the close association between lipid metabolic reprogramming and the development of lung cancer has been gradually revealed (5). A previous meta-analysis indicated that the risk of lung cancer development is positively associated with serum levels of total cholesterol, and negatively associated with total triglycerides (6). In addition, some lipid-modifying drugs, such as simvastatin, have been shown to inhibit the proliferation and metastasis of lung cancer cells by suppressing intracellular cholesterol synthesis, inducing cell cycle arrest and apoptosis, and reversing resistance to tyrosine kinase inhibitors (7, 8). Therefore, the regulation of lipid metabolism has been identified as a potential therapeutic target to improve prognosis in patients with lung cancer. Moreover, several studies have tried to construct prognostic models for lung cancer patients based on lipid metabolism-related genes. For example, Zhu’s study focused on early-stage LUAD patients using lipid metabolism-related genes to establish prognostic models and validated them according to external Gene Expression Omnibus (GEO) databases (9). Another study developed a signature based on serum lipid profiles to distinguish patients with early-stage lung cancer from healthy individuals, thereby achieving early diagnosis and treatment (10). Nevertheless, the aforementioned studies both focused on the development of diagnostic and prognostic models for early-stage LUAD. Moreover, the validity and robustness of the constructed models with a single feature are relatively poorer than those of multifeature models. Hence, deeper insight into a multifeature signature model for LUAD patients and its prognostic implications is needed.

Emerging studies have revealed that lipid metabolic reprogramming is not limited to tumor cells, as it is also closely associated with the function of immune cells infiltrating the tumor microenvironment. For instance, studies have demonstrated that enhanced lipid uptake and lipid oxidative phosphorylation are critical for tumor-associated macrophage polarization, and the lipid uptake-related molecule *CD36* has been identified as a potential tumor marker (11). Similarly, the lipid metabolism pattern of tumor-infiltrating myeloid-derived suppressor cells (T-MDSCs) also shifts to fatty acid uptake and oxidation, thereby mediating the immunosuppressive function of T-MDSCs *via* the STAT3 and STAT5 signaling pathways (12, 13). In addition, a recent study revealed a close association between the degree of lipid metabolism signaling-related mutations and the efficacy of immune checkpoint inhibitors in lung cancer patients (14). Therefore, we attempted to combine lipid metabolism-related and immune-related genes to establish a novel prognostic model for LUAD patients according to the interactions between lipid metabolism and antitumor immunity.

**Figure 1:**
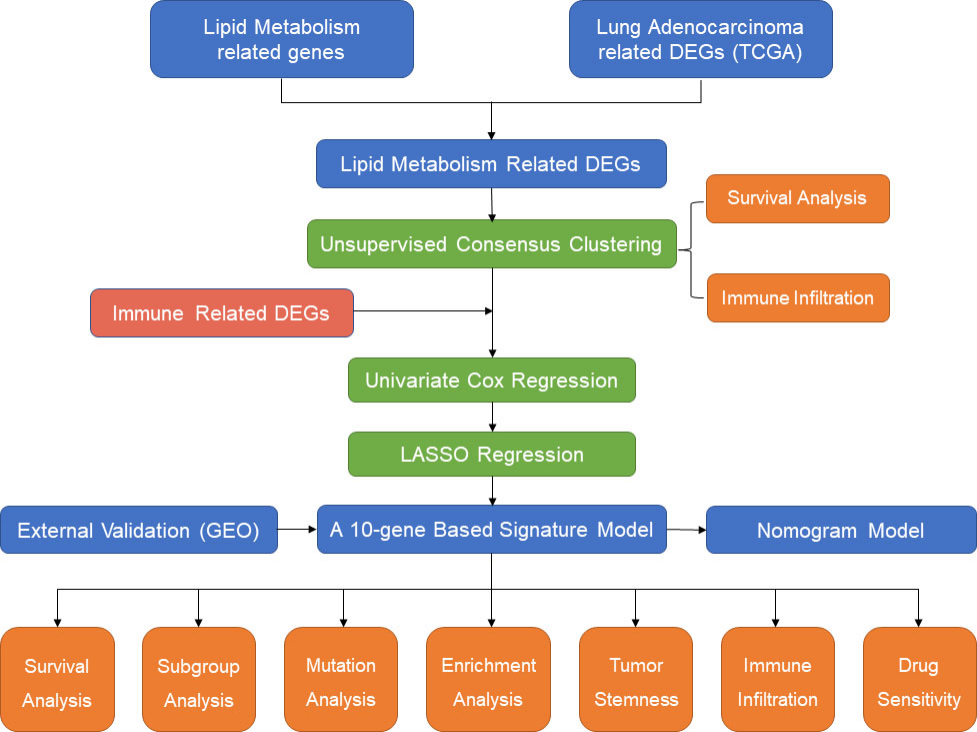
The flow diagram of the present study.

In the present study, we first identified subgroups of LUAD patients based on different lipid metabolism patterns in an unsupervised clustering approach based on The Cancer Genome Atlas (TCGA) cohort (https://www.cancer.gov/tcga/). The differences in overall survival and immune infiltration levels between different subgroups were compared. Then, lipid metabolism-related and immune-related differentially expressed genes (DEGs) were included for the establishment of the signature model by univariate Cox regression and least absolute shrinkage and selection operator (LASSO) regression. Five independent GEO datasets (15) were screened out and employed for external validation. In addition, functional enrichment analysis and somatic mutation analysis were performed to investigate the potential mechanisms of survival differences in different risk populations. Finally, we also evaluated the correlation between risk scores and immune infiltration levels and chemotherapeutic drug sensitivity. Therefore, our study provides novel insight into individualized treatment strategies and the prognostic prediction of LUAD patients from the perspective of immune-metabolic crosstalk. A flow chart summarizing the present study is shown in **Figure 1**.

## Materials and methods

### Data acquisition and preprocessing

The RNA-sequencing data and clinical information of the lung adenocarcinoma (LUAD) patients were downloaded from the TCGA data portal (https://www.cancer.gov/tcga/) and UCSC Xena browser (https://xenabrowser.net/). All of the Fragments Per Kilobase Million (FPKM) transcriptome data were log-transformed and converted to transcripts per million (TPM) before analysis.

For external validation, five independent datasets [GSE13213 (16), GSE31210 (17, 18), GSE37745 (19), GSE68465 (20), GSE72094 (21)] along with clinical information were downloaded from the GEO database (https://www.ncbi.nlm.nih.gov/geo/). The following inclusion criteria were applied for screening the qualified GEO datasets: (1) achievable datasets with complete gene expression profiles and survival information limited to LUAD patients; (2) the raw gene expression data could be downloaded in CEL files and included the corresponding probe information; and (3) the total number of samples was no less than 50. The raw matrix data were log2-transformed, quantile normalized, and averaged over duplicate genes using the ‘limma’ package in R software. The clinical characteristics of LUAD patients in the training datasets (TCGA) and the validation datasets (GEO) are displayed in **Supplementary Table S1**.

### Identification of lipid metabolism-related and immune-related differentially expressed genes

The ‘limma’ R package was applied to perform differential gene expression analysis between lung adenocarcinoma and normal tissues in the TCGA dataset. The differentially expressed genes (DEGs) with absolute fold change (|*logFC*|) > 1.5 and adjusted *p value* < 0.05 were selected. As for the genes related to lipid metabolism, we included all of the genes in 34 lipid metabolism-related gene sets from the Molecular Signature Database (MsigDB, https://www.gsea-msigdb.org/gsea/msigdb/) (22) and took the intersection to obtain the final 1996 lipid metabolism-related genes. The detailed information on the lipid metabolism-related gene sets is provided in **Supplementary Table S2**. In addition, we downloaded all of identified 2483 immune-relevant genes from the ImmPort database (https://www.immport.org/) (23). After removing duplicated genes, we finally obtained a total of 1793 immune-related genes. Then, by taking the intersection of lipid metabolism-related and immune-related genes with DEGs of LUAD, lipid metabolism-related and immune-related DEGs were obtained for subsequent analysis. The details of lipid metabolism-related and immune-related genes are presented in **Supplementary Table S3**.

### Unsupervised consensus clustering of lipid metabolism-related differentially expressed genes

To explore the prognostic impact of different lipid metabolism patterns, we adopted the ‘ConsensusClusterPlus’ R package to identify the subgroups of LUAD patients based on lipid metabolism-related DEGs. In detail, the expression profiles were clustered using a partition around medoids (PAM) approach with the Euclidean distance metric and a subsampling ratio of 0.8 for 1000 iterations. To ensure the reproducibility of our approach, we arbitrarily set the number of random seeds to 999999 in the ‘ConsensusClusterPlus’ package. Furthermore, the clustering reproducibility of the consensus clustering method was also verified in the five external GEO datasets. The optimal cluster number was determined according to the clustering consistency, cumulative distribution function (CDF) curve, the relative changes in the area under the CDF curve, and *Silhouette* clustering index. Survival curves for each cluster were analyzed with the Kaplan–Meier method. In addition, we also utilized several immune-related algorithms (‘immunedeconv’ R package) (24), including xCell (25), quanTIseq (26), and MCPcounter (27), to analyze the immune cell infiltration patterns in the tumor microenvironment, thus exploring the underlying causes of survival differences.

### Construction and validation of a prognostic signature based on lipid metabolism-related and immune-related genes

For the close association between lipid metabolism and immune infiltration patterns, we sought to construct and validate the feasibility of combining lipid metabolism-related and immune-related genes to construct a clinical prognostic signature for LUAD patients. First, univariate Cox regression (‘survival’ R package) was conducted based on lipid metabolism-related and immune-related DEGs to screen the prognosis-related genes in the TCGA training set. Then, the least absolute shrinkage and selection operator (LASSO) regression (‘glmnet’ R package) was further performed to narrow down the candidate genes and establish the prognostic signature. The risk score was calculated based on the linear combination of the regression coefficient (*β*) derived from the LASSO regression multiplied by gene expression levels. The specific calculation formula for the risk score was as follows:


$$Risk\;Score=\sum_{i=1}^{n}Coefficient\;{\left(\beta \right)}_{i}*x_{i}$$

LUAD patients were categorized into low- and high-risk groups according to the median risk score. Subsequently, Kaplan–Meier survival curves (‘survminer’ and ‘survival’) and time-dependent receiver operating characteristic (ROC) curves (‘pROC’ R package) were plotted to assess the prognostic value of the clinical model. For external validation, five independent datasets from GEO (GSE13213, GSE31210, GSE37745, GSE68465, GSE72094) were employed to verify the robustness of the novel signature, and prognostic meta-analysis (‘meta’ and ‘forestplot’ R package) was conducted to comprehensively evaluate the prognostic value based on a fixed-effects model. We further compared the expression patterns of signature genes between normal and LUAD tissues according to The Human Protein Atlas database (HPA, https://www.proteinatlas.org/, accession date: April 2022).

### Comparison of clinical characteristics and subgroup analysis based on the prognostic signature model

To further assess the correlations between the prognostic signature model and clinical characteristics of LUAD patients, differences in risk scores for each clinical feature, including age, T stage, N stage, M stage, pathological TNM stage, and primary outcome, were compared using the Wilcoxon rank-sum test or Kruskal–Wallis test. Then, we also conducted subgroup analysis according to pathological TNM stages, ages, and sexes to evaluate the prognostic significance of the signature model. Univariate and multivariate Cox regression analyses (‘survival’ R package) were also performed to identify prognostic factors independently associated with the overall survival of LUAD patients.

### Construction and evaluation of a predictive nomogram model for lung adenocarcinoma patients

Based on the results of the multivariate Cox regression analysis, we further constructed a predictive nomogram model (‘rms’ and ‘survival’ R package) for predicting the probability of 1-year, 3-year, and 5-year overall survival for LUAD patients (28). Calibration curves were generated to assess the accuracy of the nomogram. For an ideal predictive model, the predictive results are expected to fall on the 45-degree diagonal line of the calibration plot and with a higher *C*-index in the Harrell concordance test. Decision curve analysis (DCA) (29) was also performed to measure the net clinical benefits of the nomogram model.

### Mutation analysis based on the prognostic signature model

The TCGA somatic mutation data of LUAD patients were downloaded from the UCSC Xena browser (https://xenabrowser.net/). The differences in somatic mutation data between the high- and low-risk groups were analyzed and presented in the form of waterfall charts (‘maftool’ R package). Tumor mutation burden (TMB) is defined as the number of tumor mutations per megabases in each tumor sample. The corresponding TMB values were calculated by the ‘tmb’ function in the ‘maftool’ R package and log-transformed for visualization.

### Functional annotation and enrichment analyses

Lipid metabolism-related DEGs were extracted for Gene Ontology (GO) and Kyoto Encyclopedia of Genes and Genomes (KEGG) enrichment analyses (‘clusterProfiler’ R package). Gene set enrichment analysis (GSEA) was performed to assess the potential differences in biological functions between different risk groups, as defined by the C2 (c2.cp.kegg.v7.4.symbols.gmt) subset retrieved from the Molecular Signature Database (MsigDB, https://www.gsea-msigdb.org/gsea/msigdb/) (22). For GSEA, terms with an adjusted *p value* < 0.05 and a false discovery rate (*FDR*) < 0.25 were considered significant. Moreover, the gene set variation analysis (GSVA) algorithm was applied based on 50 hallmark pathways described in the Molecular Signature Database to identify enriched signaling pathways between the low-risk and high-risk groups (‘GSVA’ R package) (30).

### Tumor immune infiltration analysis

The Tumor Immune Estimation Resource algorithm (TIMER, http://timer.cistrome.org/) was employed to estimate whether the risk score was correlated with immune cell infiltration levels in the LUAD tissues (31). We also applied the xCell algorithm (25) to calculate the tumor microenvironment scores, immune scores, and stroma scores with the ‘immunedeconv’ R package (24). The DNA methylation-based stemness scores (DNAss) and RNA-based stemness scores (RNAss) of TCGA-LUAD patients were retrieved from the UCSC Xena browser (http://xena.ucsc.edu/). The immunophenoscore (IPS) of TCGA-LUAD patients was downloaded from The Cancer Immunome Database (TCIA, https://tcia.at/home) (32). The TIDE scores, dysfunction, and exclusion scores were acquired from the Tumor Immune Dysfunction and Exclusion website (TIDE, http://tide.dfci.harvard.edu/) (33). In addition, the single-sample gene set enrichment analysis (ssGSEA) (22) was performed to quantify the relative immune cell infiltration levels and immune function between the low- and high-risk groups (‘GSVA’ R package).

### Drug sensitivity analysis

For the drug sensitivity analysis, we obtained the analyzed dataset of six commonly used chemotherapeutic drugs (cisplatin, docetaxel, paclitaxel, gemcitabine, vinorelbine, and bleomycin) for lung cancer from the Genomics of Drug Sensitivity in Cancer database (GDSC, https://www.cancerrxgene.org/) (34). The ‘pRRophetic’ R package was utilized to estimate the half inhibitory concentration (IC50) values of each chemotherapeutic drug.

### Cell lines and cell culture

A549 and H1299 cell lines were provided by Stem Cell Bank, Chinese Academy of Sciences. The A549 and H1299 cells were maintained in the DMEM medium, containing 80 U/L penicillin and 0.08 mg/mL streptomycin. 10% of fetal bovine serum was also added to the medium. The cells were cultured in a conventional incubator at 37°C in a 5% CO_2_ atmosphere.

### Cell transfection

Small interfering RNA (siRNA) targeting human *PSMC1*, and negative control siRNA (siNC) were purchased from Shanghai GenePharma (Shanghai, China). The transient transfection of siRNA was performed according to the manufacturer’s instructions. First, A549 and H1299 cells were seeded the day before transfection at 30-50% confluency. siRNA duplexes were diluted into reduced serum media Opti-MEM^®^. Then, add transfection reagent lipo 3000 (Invitrogen) into the siRNA solution, vortex-mixed, and incubated for 5 min at room temperature. Finally, lipo 3000-siRNA complexes were added in a fresh medium to incubate cells at 37 °C. 24 h later, the transfected cells were collected for further experiments. The knockdown efficiency of siRNA was tested by quantitative real-time PCR assay.

### Real-time qPCR assay

The quantitative real-time PCR assay was designed to validate the efficiency of transfection. Firstly, extract total RNA from the samples with Trizol reagent. By the process of reverse transcription, the extracted RNAs were converted to cDNA. PCR amplification was carried out according to the following steps: the denaturation step lasted for 10 seconds at 95°C, the annealing step lasted for 20 seconds at 60°C, the extension step lasted for 30 seconds at 72°C, and 40 cycles were carried out in total. The primer sequences were designed as followed: *PSMC1*: F: 5’-CAGTAGCAAACCAAACCTCAGC-3’, R: 5’-TGGCGTCAATTTCATCAATAAAC-3’; *GAPDH*: F: 5’-GGAGCGAGATCCCTCCAAAAT-3’, 5’-GGCTGTTGTCATACTTCTCATGG-3’. The expression level of the *GAPDH* was taken as endogenous control, and the 2-^△△Ct^ value was used to qualify the relative gene expression levels.

### Cell proliferation assay

Cell Counting Kit-8 (CCK-8, Dojindo, Japan) was applied to assess cell proliferation ability as instructed by the manufacturer. Cells were plated into 24-well plates (6×10^4^ cells/well) for indicated time points. 10 μL CCK-8 solution was added to each well followed by incubation for another 1 hour. Lastly, the absorbance was measured at 450 nm with a microplate reader (Bio-Gene, China).

### Wound healing assay

A wound healing assay was carried out to assess cell migration. Briefly, monolayer cells were wounded by scratching the surface of each well as uniformly as possible with a sterile 200 µL pipette tip. The wells were then rinsed with phosphate-buffered saline three times and were incubated at 37°C for 48 h. Images of the initial wound, and the movement of cells into the scratched area, were captured using an inverted microscope equipped with a digital imaging system (Leica Microsystems GmbH, Wetzlar, Germany).

### Transwell migration assay

The 24-well transwell chambers (BD Biosciences, San Jose, CA, USA) with 8-μm pores were used to assess cell migration. Cells (1×10^4^ cells/well) in serum-free medium were seeded into the upper chamber. The complete growth medium was added to the lower chamber as a chemoattractant. After culturing for 24 and 48 h at 37°C, noninvasive cells in the upper chamber were removed with cotton swabs carefully, and invasive cells on the lower membrane surface were fixed in methanol and stained with 0.1% crystal violet (Sigma-Aldrich) for 15 min. Finally, the invasive cells were photographed and counted under a microscope (Nikon, Tokyo, Japan).

### Statistical analysis

R software 4.1.3 was used for data analysis and visualization. Comparisons between two groups were evaluated by the Wilcoxon rank-sum test, while the Kruskal–Wallis test was conducted to compare more than two groups. Categorical variables were compared using Fisher’s exact or Chi-square tests. The log-rank test was used to determine the difference between survival curves. Correlations between two variables were tested with the Spearman correlation test. All *p values* < 0.05 were considered statistically significant.

## Results

### Identification and exploration of lipid metabolism-related differentially expressed genes

**Figure 2:**
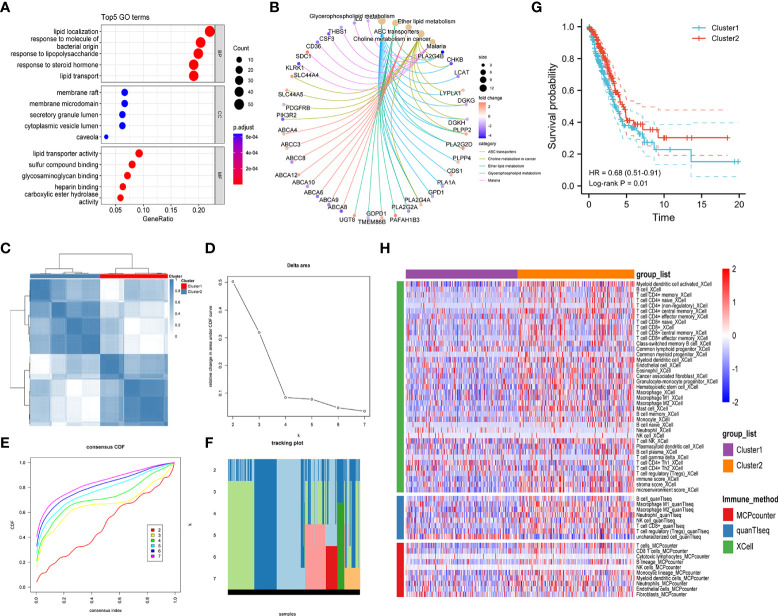
Exploration of lipid metabolism-related DEGs. Gene ontology (GO) functional annotation **(A)** and KEGG pathway enrichment analysis **(B)** of lipid metabolism-associated DEGs. **(C)** Heatmap of unsupervised consensus clustering. **(D)** The plot of changes in the relative area under the cumulative distribution function (CDF) curve from k = 2 to 7. **(E)** Consensus CDF curve plot. **(F)** The tracking plot of the clustering samples. **(G)** Kaplan–Meier curves for the overall survival of LUAD patients in different clusters. **(H)** Comparison of immune cell infiltration patterns between different clusters performed by the xCell, quanTIseq, and MCPcounter algorithms.

To investigate the prognostic significance of lipid metabolism-related genes in LUAD patients, we first obtained the differentially expressed genes (DEGs) by comparing LUAD and normal lung tissues based on the TCGA database (**Supplementary Table S4**). Then, a total of 247 common DEGs were detected as lipid metabolism-related DEGs through the intersection of DEGs of LUAD and genes associated with lipid metabolism (**Supplementary Table S5**). The biological processes and molecular function in GO annotation indicated that the DEGs were significantly enriched in lipid localization, lipid transportation, and lipid metabolism-related receptor binding, and the cell component was primarily located in cell membranes that were rich in lipids (**Figure 2A**). In addition, KEGG pathway analysis predicted that lipid metabolism-related DEGs were enriched in the pathways of glycerophospholipid metabolism, ether lipid metabolism, choline metabolism, and ABC transporters (**Figure 2B**).

Unsupervised consensus clustering analysis was further carried out to identify clusters of LUAD patients with distinct lipid metabolism patterns (**Figures 2C–F**, **Figure S1**). The results indicated that LUAD patients were divided into two clusters, and a significant overall survival difference was observed between the two clusters (hazard ratio (*HR*): 0.68, 95% confidence interval (CI): 0.51-0.91, log-rank *p value* = 0.01) (**Figure 2G**). In addition, similar consensus clustering patterns were also determined in the five GEO datasets (**Figure S2**), and the consensus clustering algorithm also well distinguished LUAD patients in the high- and low-risk groups from healthy controls (**Figure S3**).

To investigate the potential causes of survival differences, we estimated the immune cell infiltration in LUAD tissues under different lipid metabolism patterns based on the xCell, quanTIseq, and MCPcounter algorithms. The results showed that the degree of immune infiltration differed obviously between different clusters, especially in the T-cell subsets, B cells, macrophages, and granulocytes (**Figure 2H**). These results suggested that the lipid metabolism patterns were closely related to the immune infiltration and prognosis of LUAD patients, which prompted us to combine the immune-related and lipid metabolism-related genes to construct a clinical prognostic model.

### Construction of a prognostic signature incorporating immune-related and lipid metabolism-related differentially expressed genes

**Figure 3:**
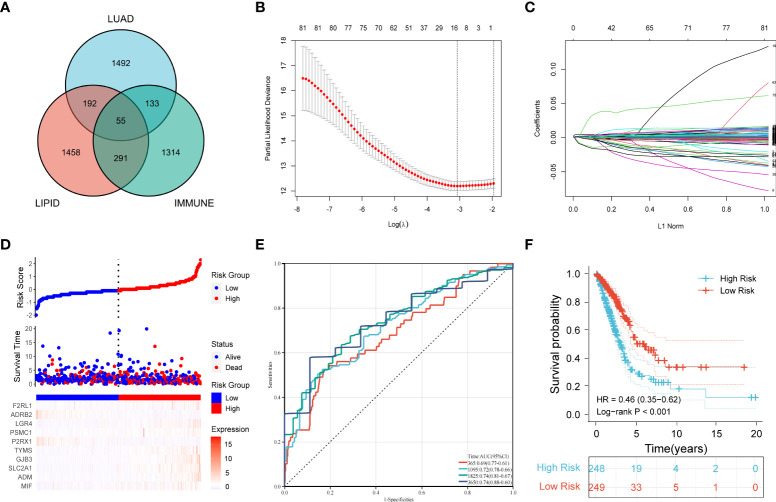
Construction of a prognostic model for LUAD patients based on immune-related and lipid metabolism-related DEGs. **(A)** The Venn diagram displays the intersection of common genes among LUAD-related DEGs and lipid metabolism-related and immune-related genes. **(B)** The LASSO regression algorithm was used to select the optimal variable (*λ*) with a 10-fold cross-validation method. **(C)** The solution path was plotted according to coefficients against the L1 norm. **(D)** The distribution of risk score, survival status, and the expression levels of coefficients in the prognostic signature. **(E)** The time-dependent ROC curves for the prognostic signature in the TCGA cohort. **(F)** The overall survival curves of LUAD patients with high- and low-risk scores were plotted based on the prognostic signature.

First, 247 lipid metabolism-related DEGs and 188 immune-related DEGs were detected by Venn diagram intersection (**Figure 3A**). Before constructing the predictive model, we performed a univariate Cox regression analysis to assess the prognostic value of these lipid metabolism-related or immune-related DEGs, and a total of 82 candidate genes with prognostic values were screened out (**Figure S4**). By using LASSO regression analysis, ten genes, *ADRB2*, *P2RX1*, *MIF*, *SLC2A1*, *F2RL1*, *PSMC1*, *LGR4*, *ADM*, *TYMS*, and *GJB3*, were selected as variables in the prognostic signature (**Figures 3B, C**). According to the median value of the signature, LUAD patients were stratified into high-risk (n = 248) and low-risk (n = 249) groups. The baseline characteristics of LUAD patients according to the predictive model are displayed in **Table 1**.

**Table 1:** Baseline characteristics and comparison of LUAD patients divided by the prognostic model.

Characteristic	levels	Low-risk	High-risk	*p*	Method
n		249	248		
event, n (%)	Alive	181 (72.7%)	136 (54.8%)	< 0.001	Chi-square
	Dead	68 (27.3%)	112 (45.2%)		
Age, n (%)	<65	103 (41.4%)	111 (44.8%)	0.501	Chi-square
	≥65	146 (58.6%)	137 (55.2%)		
T Stage, n (%)	T1	104 (41.8%)	62 (25.0%)	< 0.001	Fisher’s test
	T2	121 (48.6%)	146 (58.9%)		
	T3	13 (5.2%)	30 (12.1%)		
	T4	9 (3.6%)	9 (3.6%)		
	Tx	2 (0.8%)	1 (0.4%)		
N Stage, n (%)	N0	178 (71.5%)	143 (57.7%)	0.002	Fisher’s test
	N1	37 (14.9%)	57 (23.0%)		
	N2	25 (10.0%)	44 (17.8%)		
	N3	1 (0.4%)	1 (0.4%)		
	Nx	8 (3.2%)	3 (1.2%)		
M Stage, n (%)	M0	239 (96.0%)	233 (94.0%)	0.256	Fisher’s test
	M1	9 (3.6%)	15 (6.0%)		
	Mx	1 (0.4%)	0 (0%)		
Stage, n (%)	Stage I	155 (62.2%)	112 (45.2%)	< 0.001	Fisher’s test
	Stage II	54 (21.7%)	69 (27.8%)		
	Stage III	29 (11.6%)	52 (21.0%)		
	Stage IV	10 (4.0%)	15 (6.0%)		
	Unknown	1 (0.4%)	0 (0%)		
Gender, n (%)	Female	145 (58.2%)	124 (50.0%)	0.080	Chi-square
	Male	104 (41.8%)	124 (50.0%)		
Outcome, n (%)	CR	168 (67.5%)	138 (55.6%)	0.021	Fisher’s test
	PD	26 (10.4%)	42 (16.9%)		
	PR	4 (1.6%)	1 (0.4%)		
	SD	18 (7.2%)	19 (7.7%)		
	Unknown	33 (13.3%)	48 (19.4%)		
time, median (IQR)		724 (476, 1268)	596.5 (332.25, 945.5)	< 0.001	Wilcoxon

Compared with the low-risk group, the proportion of LUAD patients who died was significantly higher in the high-risk group (**Figure 3D**). To evaluate the predictive accuracy of the prognostic signature, time-dependent ROC and Kaplan–Meier curves were plotted and compared. The results showed that the areas under the ROC curves (AUCs) for 1-, 3-, 5-, and 10-year overall survival were 0.69, 0.72, 0.74, and 0.74, respectively, in the TCGA cohort (**Figure 3E**). Kaplan–Meier analysis confirmed that LUAD patients in the low-risk group had significantly longer overall survival than those in the high-risk group (*HR*: 0.46, 95% CI: 0.35-0.62, log-rank *p value* < 0.001) (**Figure 3F**).

**Figure 4:**
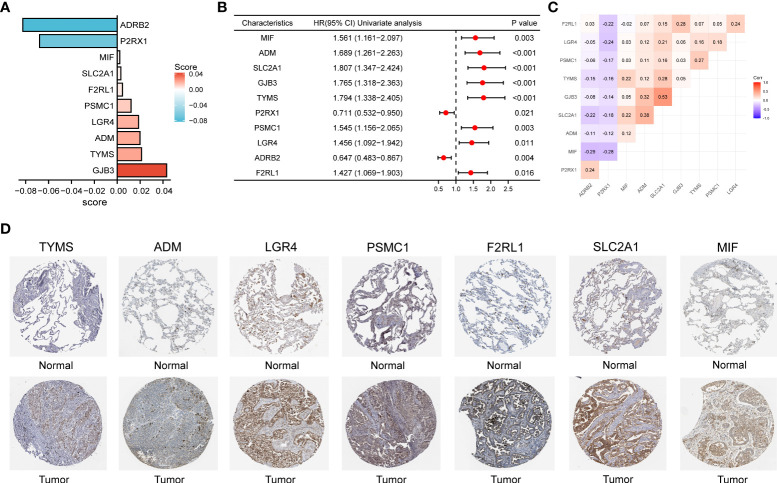
**(A)** The bar charts display the variables and corresponding coefficients in the prognostic signature. **(B)** The forest plot shows the results of hazard ratios and 95% confidence intervals of signature genes from the univariate Cox regression analysis. **(C)** The correlation matrix illustrates the correlations between genes involved in the prognostic signature. **(D)** Representative immunohistochemical staining images of *TYMS* (antibody HPA074922, 10×), *ADM* (antibody CAB016075, 10×), *LGR4* (antibody HPA030267, 10×), *PSMC1* (antibody HPA016885, 10×), *F2RL1* (antibody CAB012989, 10×), *SLC2A1* (antibody CAB002759, 10×), and *MIF* (antibody CAB005284, 10×) in normal and LUAD tissues are retrieved from The Human Protein Atlas database (HPA, https://www.proteinatlas.org/, accession date: April 2022). It should be noted that the immunohistochemistry staining of *ADRB2*, *P2RX1*, and *GJB3* were absent from the HPA database.

By plotting the bar charts of variables (**Figure 4A**) and the forest plot of univariate Cox regression analysis (**Figure 4B**), we found that two genes (*ADRB2* and *P2RX1*) involved in the prognostic signature were protective factors (*HR* < 1), while the other eight genes were risk factors (*HR* > 1) for LUAD patients. The correlation matrix revealed that the expression levels of the two protective genes showed a significant positive correlation with each other, but a negative correlation with the other eight risk genes in expression levels (**Figure 4C**). To further validate the expression patterns of the signature genes in LUAD patients, we have compared the protein expression profiles determined by immunohistochemistry staining which are available in the HPA database. The results indicated that seven risk genes (*TYMS*, *ADM*, *LGR4*, *PSMC1*, *F2RL1*, *SLC2A1*, and *MIF*) of the signature were overexpressed in the LUAD tissues compared to the normal tissues (**Figure 4D**).

### Validation of the prognostic signature based on the GEO database

To further confirm the robustness of the prognostic signature, five GEO datasets were screened and enrolled for external validation cohorts. The risk scores of each GEO dataset were calculated, and LUAD patients were divided into high-risk and low-risk groups according to the median cutoff value of risk scores. The survival analysis of the five validation datasets all demonstrated that LUAD patients in the high-risk group had a significantly poorer overall survival than those in the low-risk groups (GSE13213: *p* = 0.003, GSE31210: *p* < 0.001, GSE37745: *p* < 0.001, GSE68465: *p* < 0.001, GSE72094: *p* < 0.001). The time-dependent ROC curves also indicated similar results to those of the TCGA training dataset (**Figures S5A–E**). Moreover, we performed a prognostic meta-analysis combining statistical outcomes from both the training and validation datasets, and the results showed that the clinical predictive signature was an independent prognostic factor for LUAD patients (*HR*: 0.44, 95% CI: 0.38-0.52, *p value* < 0.001), as shown in **Figure S5F**.

### Correlation analysis between the prognostic signature and clinical characteristics of LUAD patients

**Figure 5:**
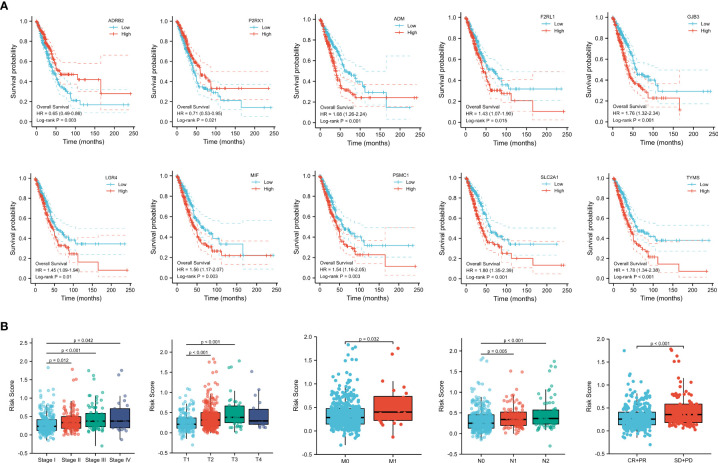
**(A)** Survival analysis of genes involved in the prognostic signature. **(B)** Subgroup analysis based on the clinical characteristics of LUAD patients.

To understand the clinical relevance and prognostic value of the signature, we first plotted survival curves to evaluate the prognostic value of each gene involved in the predictive signature. Through the log-rank test, patients in the high *ADM*, *F2RL1*, *GJB3*, *LGR4*, *MIF*, *PSMC1*, *SLC2A1*, and *TYMS* expression groups had worse overall survival outcomes than those in the low expression groups (*p* < 0.05). In contrast, LUAD patients with high expression of *ADRB2* and *P2RX1* had significantly longer overall survival than those in the low-expression group (*p* < 0.05), as shown in **Figure 5A**. In addition, subgroup analysis based on clinical features suggested that the levels of risk score varied significantly among the pathological stage (Stage II and Stage III and Stage IV vs. Stage I, *p* < 0.05), T stage (T2 and T3 vs. T1, *p* < 0.05), M stage (M1 vs. M0, *p* < 0.05), N stage (N1 and N2 vs. N0, *p* < 0.05), and primary outcome (CR+PR vs. SD+PD, *p* < 0.05), as shown in **Figure 5B**.

**Figure 6:**
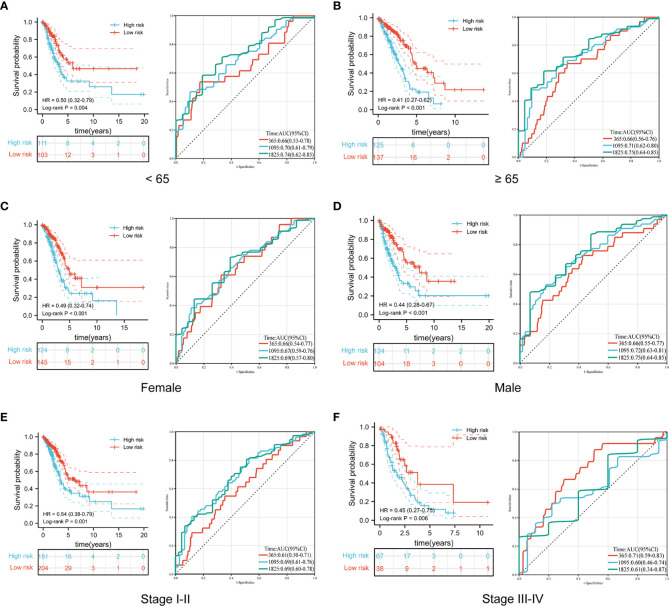
Comparison of overall survival and time-dependent ROC curves for LUAD patients of different ages **(A, B)**, sexes **(C, D)**, and pathological stages **(E, F)** between the high-risk and low-risk groups.

To further explore the predictive value of the prognostic signature, we conducted a subgroup analysis of LUAD patients in the high- and low-risk groups based on different clinical features. Similar to the results in the training and validation cohorts, LUAD patients in the high-risk group with different clinical features all exhibited poorer survival than those in the low-risk group. Moreover, time-dependent ROC curves indicated that the prognostic signature had a comparable predictive ability at 1, 3, and 5 years for patients of different ages (**Figures 6A, B**). Furthermore, it performed better in predicting survival for males (**Figures 6C, D**) and patients with early-stage disease (**Figures 6E, F**).

### Construction and evaluation of a nomogram for survival prediction in LUAD patients

To establish a prognostic nomogram for predicting the survival of LUAD patients, univariate and multivariate Cox analyses were carried out using clinical features and risk scores. Univariate Cox regression indicated that pathological stage, T stage, N stage, M stage, primary outcome, and risk score were closely correlated with the overall survival of LUAD patients. Further multivariate analysis confirmed that the primary outcome and risk score were independent factors affecting the prognosis of LUAD patients (**Table 2**).

**Table 2:** The univariate and multivariate Cox regression analyses of clinical characteristics for overall survival in LUAD patients.

Characteristic	Total(N)	Univariate analysis		Multivariate analysis	
		Hazard ratio (95% CI)	*p*-value	Hazard ratio (95% CI)	*p*-value
**Age**	497				
<65	214	Reference			
>=65	283	1.078 (0.800-1.453)	0.623		
**T Stage**	497				
T1	166	Reference			
T2	267	1.481 (1.034-2.120)	**0.032**	0.904 (0.617-1.323)	0.603
T3	43	2.956 (1.739-5.024)	**<0.001**	1.241 (0.640-2.404)	0.523
T4	18	2.975 (1.529-5.786)	**0.001**	1.274 (0.581-2.796)	0.545
Tx	3	4.745 (1.140-19.755)	**0.032**	0.431 (0.033-5.646)	0.522
**N Stage**	497				
N0	321	Reference			
N1	94	2.439 (1.727-3.445)	**<0.001**	1.419 (0.795-2.531)	0.236
N2	69	3.108 (2.119-4.559)	**<0.001**	1.654 (0.649-4.214)	0.292
N3	2	0.000 (0.000-Inf)	0.994	0.000 (0.000-Inf)	0.995
Nx	11	1.656 (0.606-4.524)	0.325	2.033 (0.490-8.423)	0.328
**M Stage**	497				
M0	472	Reference			
M1	24	2.210 (1.300-3.758)	**0.003**	0.263 (0.016-4.426)	0.354
Mx	1	0.000 (0.000-Inf)	0.994	0.000 (0.000-Inf)	0.998
**Pathological Stage**	497				
Stage I	267	Reference			
Stage II	123	2.349 (1.635-3.375)	**<0.001**	1.582 (0.850-2.943)	0.148
Stage III	81	3.528 (2.403-5.181)	**<0.001**	1.730 (0.637-4.700)	0.282
Stage IV	25	3.862 (2.224-6.707)	**<0.001**	7.003 (0.378-129.642)	0.191
Unknown	1	0.000 (0.000-Inf)	0.995		
**Gender**	497				
Male	228	Reference			
Female	269	0.954 (0.711-1.279)	0.752		
**Outcome**	497				
CR	306	Reference			
PR	5	2.533 (0.621-10.339)	0.195	3.412 (0.786-14.812)	0.101
SD	37	1.133 (0.568-2.258)	0.723	1.037 (0.516-2.085)	0.918
PD	68	4.136 (2.870-5.960)	**<0.001**	3.541 (2.394-5.239)	**<0.001**
Unknown	81	3.595 (2.464-5.243)	**<0.001**	3.373 (2.255-5.047)	**<0.001**
**Risk score**	497				
Low	249	Reference			
High	248	2.172 (1.605-2.939)	**<0.001**	1.832 (1.331-2.521)	**<0.001**

P values < 0.05 are indicated in bold.

**Figure 7:**
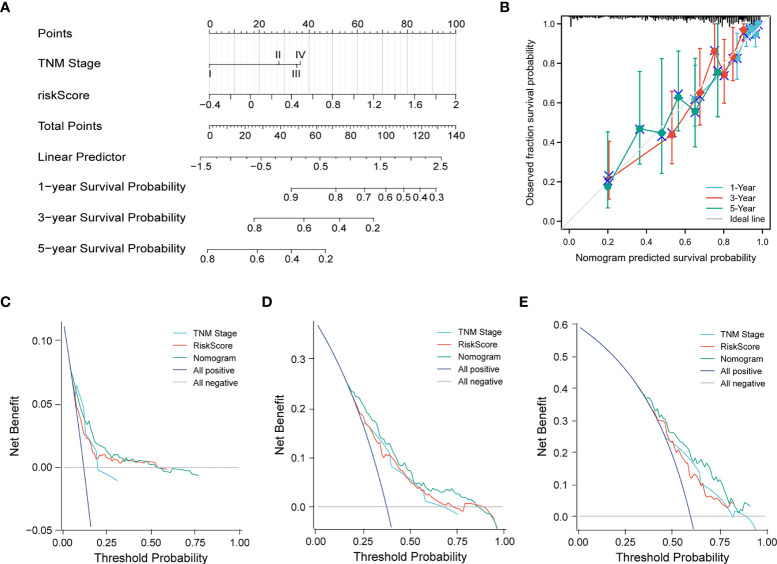
**(A)** A nomogram model was constructed to predict the 1-year, 3-year, and 5-year overall survival of LUAD patients. **(B)** Calibration curves of the nomogram model for 1-year, 3-year, and 5-year overall survival. **(C–E)** Decision curve analysis for 1-year **(C)**, 3-year **(D)**, and 5-year **(E)** overall survival of the nomogram model.

Subsequently, a nomogram incorporating independent factors was established for predicting the 1-year, 3-year, and 5-year overall survival (**Figure 7A**). Further analyses of the performance of the nomogram model showed that the *C*-index was 0.726, and the calibration curves fit well with the ideal diagonal line. These findings indicate good discrimination of the model (**Figure 7B**). Decision curve analysis also demonstrated that compared with the TNM stage or prognostic signature, the nomogram model had better performance for predicting the 1-year, 3-year, and 5-year overall survival of LUAD patients (**Figures 7C–E**). In addition, we also performed a horizontal comparison of the *C*-indices of nomogram models based on the TCGA-LUAD database in other similar studies. The results indicated that our study exhibits certain advantages in the consistency of model fitting compared with previously reported models constructed with a single characteristic, as shown in **Table 3**.

**Table 3:** Comparison with the *C*-indices of other previously reported nomogram models in the TCGA-LUAD cohort.

Literature	Characteristics	*C*-index
Present study	Lipid metabolism and immune	0.726
Jin_Duan_2021 (35)	Autophagy	0.721
Zetian_Gong_2022 (36)	Pyroptosis	0.711
Xuelong_Wang_2021 (37)	Methylation	0.710
Chunyu_Li_2020 (38)	None	0.710
Lulu_He_2020 (39)	Metabolism	0.702
Jian_Yang_2022 (40)	Cell cycle checkpoints	0.700

### Mutation analysis based on the prognostic signature

**Figure 8:**
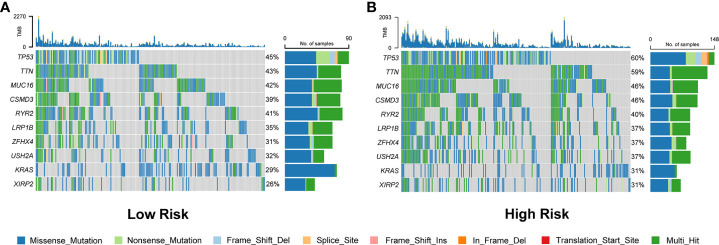
Comparison of somatic mutation rates between the low-risk **(A)** and high-risk **(B)** groups in the TCGA cohort.

Waterfall plots were used to display the type and frequency of somatic mutations in the high-risk and low-risk groups. These data suggested that the overall levels of tumor mutation burden were significantly higher in the high-risk group than those in the low-risk group (**Figure 8** and **Figure S6A**). In addition, compared with the low-risk group, the most frequently mutated genes, *TP53* and *TTN*, had a significantly higher frequency of mutations in the high-risk group. The other eight LUAD-mutated genes (*MUC16*, *CSMD3*, *RYR2*, *LRP1B*, *ZFHX4*, *USH2A*, *KRAS*, and *XIRP2*) also showed various degrees of increasing trends (**Figure 8**).

### Functional enrichment analysis

**Figure 9:**
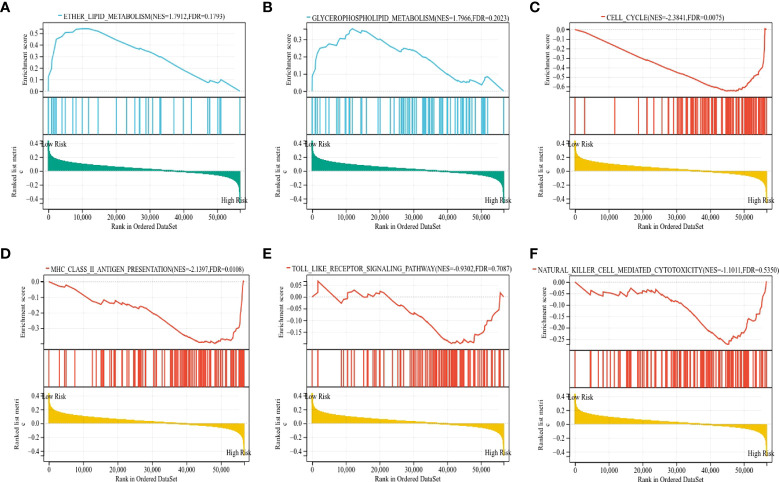
The KEGG signaling pathways enriched by GSEA. **(A, B)** The signaling pathways of ether lipid metabolism and glycerophospholipid metabolism were mainly enriched in the low-risk group. **(C–F)** The signaling pathways of the cell cycle, MHC class II antigen presentation, Toll-like receptor, and natural killer cell-mediated cytotoxicity were mainly enriched in the high-risk group.

To gain insight into the underlying mechanisms of the survival difference, gene set enrichment analysis (GSEA) was performed. The results revealed that lipid metabolism-related pathways, including ether lipid metabolism and glycerophospholipid metabolism, were mainly enriched in the low-risk group (**Figures 9A, B**). Moreover, immune-related pathways, including the cell cycle, major histocompatibility complex (MHC) class II antigen presentation, Toll-like receptor, and natural killer cell-mediated cytotoxicity, were mainly enriched in the high-risk group (**Figures 9C–F**).

To further distinguish the differences in biological behaviors between the high-risk and low-risk groups of LUAD patients, gene set variation analysis (GSVA) was carried out. The results demonstrated that pathways associated with tumor progressions, such as glycolysis, the G2/M checkpoint, Myc targets, PI3K-AKT-mTOR, DNA repair, hypoxia, and epithelial-mesenchymal transition, were mainly enriched in the high-risk group of LUAD patients. Moreover, inflammatory and immune-related signaling pathways, including TNF-α, TGF-β, and interferon-γ, were also enriched in the high-risk group. In contrast, metabolism-related pathways, such as bile acid metabolism, myogenesis metabolism, and heme metabolism, were significantly enriched in the low-risk groups (**Figures S7A, B**).

### Immune infiltration analysis based on the prognostic signature

**Figure 10:**
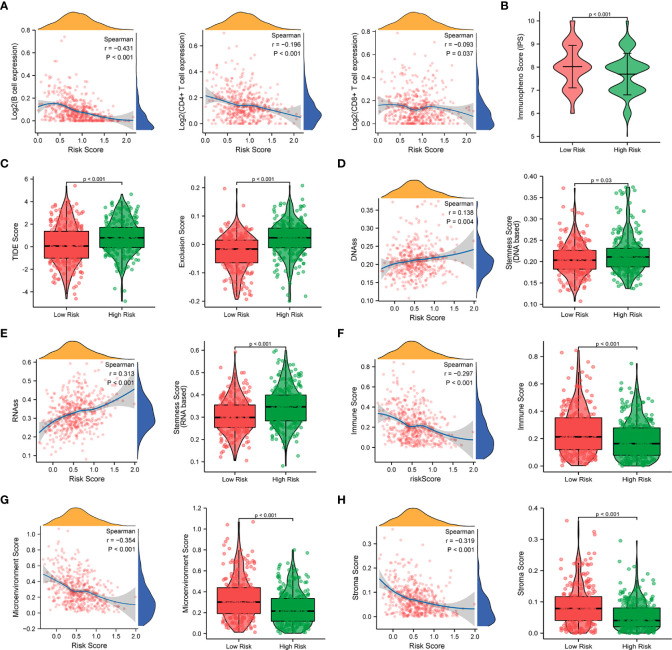
Correlation analysis of the prognostic signature and immune infiltration in LUAD patients. **(A)** Correlation analysis of the risk score and immune infiltration *via* The Tumor Immune Estimation Resource (TIMER, http://timer.cistrome.org/) database. **(B, C)** Comparison of the IPS score **(B)** and the TIDE score **(C)** between the high-risk and low-risk groups. **(D–H)** Correlations between risk score and DNA methylation-based stemness score **(D)**, RNA-based stemness score **(E)**, immune score **(F)**, microenvironment score **(G)**, and stroma score **(H)**, and the comparison of various scores between the high-risk and low-risk groups.

Since a close association between the prognostic signature and immune response was found in the functional enrichment analysis, we further investigated the correlation between the risk scores and infiltrated immune cells. The results of the TIMER database showed that the risk score was negatively correlated with B cells (*r* = -0.431, *p* < 0.001), ${\text{CD}}_{4}^{+}$ T cells (*r* = -0.196, *p* < 0.001), and ${\text{CD}}_{8}^{+}$ T cells (*r* = -0.093, *p* = 0.037), as shown in **Figure 10A**. Compared with the low-risk group, the high-risk group displayed substantially lower IPS scores and markedly higher TIDE scores, dysfunction scores, and exclusion scores (*p* < 0.001), which indicate poorer efficacy of immune checkpoint blockade therapy (**Figures 10B, C**, **Figure S6B**). We next evaluated the tumor stemness between different risk patterns using DNAss and RNAss. The results indicated that the risk score was positively correlated with values of the DNAss (*r* = 0.138, *p* = 0.004) and RNAss (*r* = 0.313, *p* < 0.001), as shown in **Figures 10D, E**. The DNAss and RNAss were also significantly higher in the high-risk group (*p* < 0.05). In addition, a negative correlation was observed between the risk score and immune score (*r* = -0.297, *p* < 0.001), microenvironment score (*r* = -0.354, *p* < 0.001), and stroma score (*r* = -0.319, *p* < 0.001), and these findings were consistent with the comparison of score values in different risk groups (*p* < 0.001), as shown in **Figures 10F–H**.

**Figure 11:**
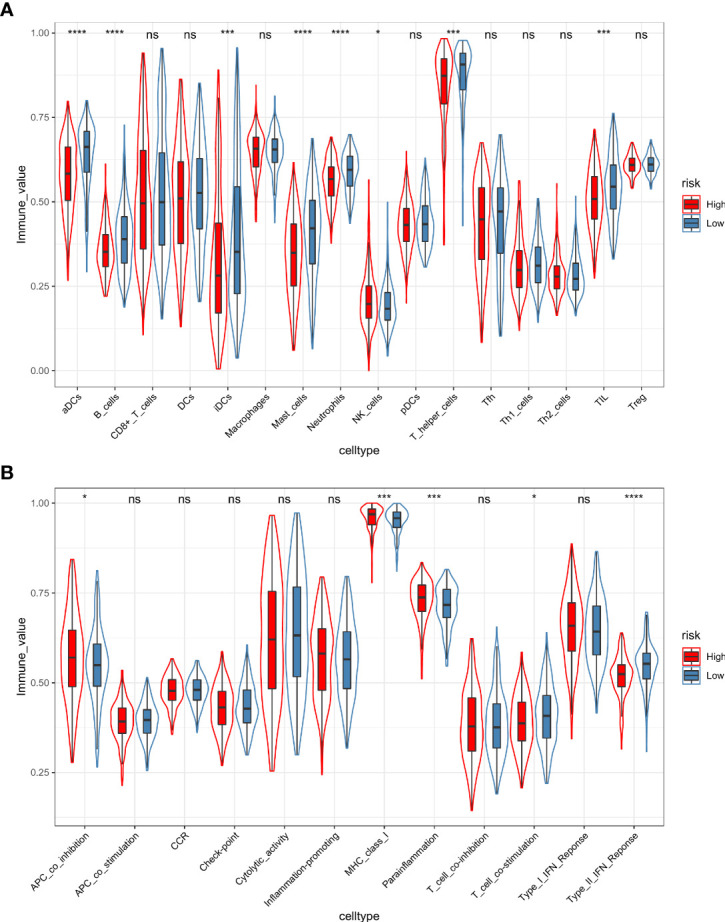
The ssGSEA algorithm was employed to quantify the immune cell infiltration **(A)** and immune function **(B)** between the high-risk and low-risk groups. *, *p value* < 0.05; ***, *p value* < 0.001; ****, *p value* < 0.0001; ns, not significant, *p value* > 0.05.

Furthermore, the ssGSEA algorithm was carried out to assess the differences in immune status between different risk groups. For immune cell type analysis, LUAD patients in the low-risk group were found to have higher infiltration of active dendritic cells (aDCs), B cells, immature dendritic cells (iDCs), mast cells, neutrophils, T helper cells, and tumor-infiltrating lymphocytes (TILs) in the tumor microenvironment. In contrast, relatively low infiltration of natural killer cells was detected in the low-risk group (*p* < 0.05). In addition, the levels of antigen-presenting cell (APC) coinhibition, MHC class I, and parainflammation were observed to be significantly higher in the high-risk group. Conversely, opposite trends were detected in the function of T-cell costimulation and Type II interferon response (*p* < 0.05) (**Figures 11A, B**).

### Chemotherapeutic drug sensitivity analysis according to the prognostic signature

To further examine the relationship between drug sensitivity and risk score, we compared the sensitivity of different risk groups of lung cancer patients to common chemotherapeutic drugs based on the GDSC database. The results revealed that the estimated IC50 values of six chemotherapeutic drugs (cisplatin, docetaxel, paclitaxel, gemcitabine, vinorelbine, and bleomycin) were significantly higher in the low-risk group (*p* < 0.05), which indicated that LUAD patients in the high-risk group were more sensitive to chemotherapy (**Figures S8A–F**).

### Downregulated *PSMC1* inhibits the proliferation and migration of lung adenocarcinoma cells

**Figure 12:**
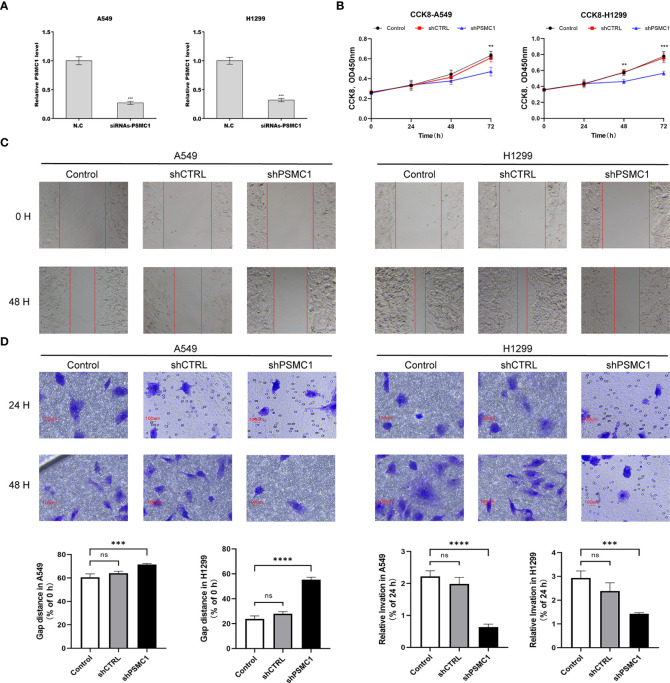
Downregulation of *PSMC1* inhibits the proliferation and migration of LUAD cells. **(A)** The quantitative real-time PCR was performed to validate the transfected efficiency. **(B)** CCK-8 assay was used to determine the proliferation ability of A549 and H1299. **(C)** The cell migration ability was analyzed using the wound-healing assay. **(D)** The cell migration ability was detected by the Transwell migration assay. Data represent mean ± SD from three replicates of each sample. ns, no significance; ^**^ *p* < 0.01; ^***^ *p* < 0.001; ^****^ *p* < 0.0001.

To further verify the value of our prognostic model, we selected the potential oncogene *PSMC1*, which has not been previously reported in LUAD patients, and confirmed its biological function through *in vitro* experiments. For the loss-of-function assay, siRNA of *PSMC1* were transfected into A549 and H1299 cells to explore whether *PSMC1* exerts effects on LUAD cell function. Firstly, the knockdown efficiency of the si-RNA was verified by RT-qPCR (**Figure 12A**). As illustrated in **Figure 12B**, the CCK-8 assay revealed that knockdown of *PSMC1* inhibited cell proliferation. In addition, both the wound-healing assay and the Transwell migration assay indicated that the migration ability decreased following *PSMC1* silencing (**Figures 12C, D**). Therefore, these findings suggested that *PSMC1* could promote the proliferation and migration of LUAD cells, which may contribute to the poor prognosis in LUAD patients to a certain extent.

## Discussion

To the best of our knowledge, the high mortality rate of lung adenocarcinoma remains the most troubling issue for clinicians (41). Hence, the exploration of an effective and robust prognostic model is imperative to inform clinical decision-making. In recent years, accumulating numbers of studies have demonstrated that lipid metabolism in the tumor microenvironment not only regulates the proliferation and invasion of tumor cells but also reshapes the function of stromal cells, especially immune cells that contribute to tumor metastasis (42). Therefore, it is suggested that there is a close link between lipid metabolism patterns and antitumor immunity. However, most previous similar studies (9, 14, 43) have constructed prognostic models based on single lipid metabolism features, which often have the limitation of poor robustness and extrapolation. To overcome the shortcomings of previous studies, both immune-related and lipid metabolism-related genes were included in the present study to further improve the accuracy and robustness of prognostic signatures by providing multiscale clinical features.

First, lipid metabolism-related DEGs were obtained by taking the intersection of the lipid metabolism-related genes and DEGs associated with LUAD. GO annotation and KEGG enrichment analyses indicated that these DEGs were mainly involved in the biological processes of lipid biosynthesis, metabolism, and transportation. Then, two main subgroups were identified using unsupervised consensus clustering based on lipid metabolism-related DEGs. Notably, significant differences in overall survival were observed between LUAD patients with different lipid metabolism patterns. Furthermore, according to different immune infiltration algorithms, we found that the infiltration patterns of immune cells varied in different subgroups, and a high infiltration level of immune cells showed a significant positive relationship with the overall survival of LUAD patients. Thus, we speculated that survival differences under different lipid metabolism patterns may be related to the immune infiltration of the tumor microenvironment. This prompted us to integrate both lipid metabolism and immune features to construct a clinical prognostic model in the present study.

By performing univariate and LASSO penalized Cox regression, 10 variables were screened out and included in the final prognostic signature. Interestingly, of these 10 variables, four (*F2RL1*, *ADRB2*, *LGR4*, *PSMC1*) were immune-related genes, four (*P2RX1*, *TYMS*, *GJB3*, *SLC2A1*) were lipid metabolism-related genes, and the two (*ADM*, *MIF*) were closely associated with both lipid metabolism and immunity. These findings suggest that immunity and lipid metabolism contributed comparable weights in the model construction. Moreover, *ADRB2* and *P2RX1* showed a negative correlation with risk scores and overall survival, while the remaining eight genes were positively correlated with these parameters. Based on the Kaplan–Meier survival curves, a significantly shorter overall survival was observed in LUAD patients in the high-risk subgroup than in those in the low-risk subgroup (*HR*: 0.46, log-rank *p value* < 0.001). The time-dependent ROC curve analysis indicated high accuracy of the prognostic signature in predicting the survival of LUAD patients. Moreover, the results of external validation based on five GEO datasets and prognostic meta-analysis all confirmed the relatively better robustness of the prognostic signature compared to previous studies (9, 37).

*F2RL1*, also known as protease-activated receptor 2 (*PAR2*), has been reported to be associated with the occurrence and development of lung adenocarcinoma (44) and intestinal tumors (45, 46). A recent study revealed that hypomethylation of the *F2RL1* promoter could upregulate its expression and promote the proliferation, migration, and invasion of LUAD cells (44). The *ADRB2* gene encodes the beta2-adrenergic receptor (*β*2-AR), which plays a crucial role in facilitating bronchodilation (47). Studies have shown that targeting *ADRB2* could enhance the sensitivity of lung cancer cells to VEGFR2-TKIs (48), but the protective mechanism of *ADRB2* on LUAD has not been mentioned in the literature. The binding of leucine-rich repeat-containing G-protein-coupled receptor 4 (*LGR4*) to its receptor R-spondin was reported to promote the proliferation and metastasis of cancer cells. Moreover, *LGR4* inhibition could improve the clinical efficacy of immune checkpoint blockade by modulating TAM polarization and ${\text{CD}}_{8}^{+}$ T-cell infiltration (49). For the lipid metabolism-related genes, thymidylate synthetase (*TYMS*) (50–52) and *SLC2A1* (53–55) have repeatedly emerged as variables in clinical prognostic signatures for LUAD patients, suggesting that they are critical metabolism-related genes that affect the prognosis of LUAD patients. A meta-analysis demonstrated that as a pyrimidine metabolic rate-limiting enzyme (56), *TYMS* expression was negatively correlated with response rate, overall survival, and progression-free survival in NSCLC patients treated with pemetrexed-based chemotherapy (57). Downregulation of *SLC2A1* can interfere with glycolysis in lung cancer cells. Thus, it can inhibit cell proliferation, migration, and the cell cycle and promote apoptosis (58, 59). In addition, for the intersected genes of two features, tumor-secreted adrenomedullin (*ADM*) is recognized as a type of regulatory polypeptide driving both tumor and lymph node angiogenesis. Therefore, it may serve as a potential target for suppressing the lymphatic metastasis of lung cancer (60). Similar to the aforementioned *TYMS* and *SLC2A1*, macrophage migration inhibitory factor (*MIF*) has also been involved in the establishment of prognostic models for LUAD patients on several independent occasions (61, 62). A previous study suggested that *MIF* overexpression could promote the Warburg effect of lung cancer cells *via* the NF-κB/HIF-1α signaling pathway, thus contributing to the progression of lung cancer (63). However, the exact mechanisms by which *PSMC1*, *P2RX1*, and *GJB3* are involved in the progression of lung cancer have not yet been reported in the literature and warrant further exploration. For further validation, we explored the role of *PSMC1* in the progression of lung cancer through *in vitro* experiments. The results have demonstrated that downregulation of *PSMC1* could attenuate the proliferation and migration of LUAD cells, which is in agreement with previous bioinformatic predictions.

The results of clinical feature-based subgroup analyses indicated good agreement between the risk scores and the disease stage. The follow-up subgroup analyses based on different ages, sexes, and TNM stages also suggested a robust predictive power of the signature in each category. Moreover, the prognostic value of the signature for LUAD patients was further confirmed by univariate and multivariate analyses. Then, a nomogram model was built based on this signature to determine individualized prognostic scores and was evaluated by the *C*-index and the calibration curves. Decision curve analysis also revealed the great clinical application value of the nomogram model, which further supported the reliability of this signature.

For a deeper investigation of the potential mechanisms affecting the survival difference, we first compared the genes with high-frequency rates of somatic mutations in lung cancers, such as *TP53*, *TTN*, and *MUC16*. The results showed that the mutation rates of *TP53* and *TTN* were highly associated with the risk scores predicted by the prognostic signature, which may be one of the reasons for the poorer prognosis of LUAD patients in the high-risk group. Through GSEA and GSVA, we found that in addition to the glycolysis, cell cycle, hypoxia, and angiogenesis phenotypes associated with tumor proliferation and invasion, several inflammatory/immune responses-related signaling pathways were also enriched in the high-risk group. These findings indicated that the survival differences may be driven by the varied immune status of LUAD patients.

To further elucidate the underlying immune-related mechanisms of the signature for predicting the prognosis of LUAD patients, a deconvolution algorithm of TIMER was employed to assess the relationship between the risk score and the immune infiltration levels. The results showed negative correlations between the risk score and several antitumor immune effector cells, particularly B cells and ${\text{CD}}_{4}^{+}$ T cells. These findings indicate a lower level of antitumor immune response in the high-risk groups. The immunophenoscore (IPS) is a tumor immunogenicity index calculated based on four representative immune cells: activated ${\text{CD}}_{4}^{+}$ T cells, activated ${\text{CD}}_{8}^{+}$ T cells, effector memory ${\text{CD}}_{4}^{+}$ T cells, and MDSCs, which is positively correlated with the response rate to immune checkpoint inhibitors (32). Conversely, the TIDE score is based on cytotoxic T lymphocyte function, and it is negatively correlated with clinical response to immune checkpoint blockade (ICB) and overall survival (33). Both IPS and TIDE algorithms (TIDE score, dysfunction score, and exclusion score) indicated that LUAD patients in the high-risk group presented with a relatively lower sensitivity to immune checkpoint inhibitors. Moreover, tumor hypoimmunogenicity and hyporesponsiveness to ICB were also corroborated by the xCell algorithm-derived immune scores, stroma scores, and microenvironment scores. Tumor stemness-related scores, such as RNAss and DNAss, are positively correlated with tumor proliferation, invasion, metastasis, and chemoresistance (64). Our findings confirmed significant positive correlations between DNAss and RNAss and the risk score, revealing the potential reason for poor prognosis in the high-risk group from another perspective.

By quantifying immune cell infiltration and immune function based on the ssGSEA algorithm, we further confirmed obvious differences in the degree of B- and T-cell subset infiltration, antigen presentation, T-cell costimulatory function, and inflammatory response between the high- and low-risk groups. These findings were consistent with the results of the GSEA and TIMER analyses described above. In addition, the infiltration levels of active dendritic cells (aDCs) and immature dendritic cells (iDCs) were higher in the low-risk group. As specialized antigen-presenting cells, DCs play a critical role in the activation of antitumor-associated T cells (65). Studies have shown that lung cancer can dynamically exclude functional DCs from tumor tissues and recruit immunosuppressive plasma DCs to the tumor microenvironment, thereby impeding immune clearance (66). These findings partially explain the poorer prognosis in the high-risk group. It is still noteworthy that the infiltration levels of mast cells and neutrophils were substantially elevated in the low-risk group. Previous studies have shown that mast cells are an important source of VEGF and can promote tumor proliferation and angiogenesis (67, 68). However, the latest research described the heterogeneity of mast cells and confirmed that the ${\text{CD}}_{103}^{+}$ mast cell subset exhibited a stronger expression of antigen presentation-related molecules including ICAM-1, CD80, and MHC-class II. This, in turn, effectively activated ${\text{CD}}_{4}^{+}$ T cells. The study also indicated that lung cancer patients with high mast cell infiltration in the tumor microenvironment exhibited better overall and disease-free survival (69). In addition, it has been reported that MHC protein is one of the key molecules in presenting antigens and activating T lymphocytes for anti-tumor immunity (70). Downregulation of MHC molecules is usually closely associated with ICB resistance and poor clinical outcomes (71, 72). Nevertheless, a recent study has demonstrated that the common MHC class I component β2-microglobulin can bind to the inhibitory receptor LILRB1 of macrophages, thereby protecting tumor cells from phagocytosis and promoting resistance to immunotherapy (73). This might partly explain the underlying causes of the enrichment of the MHC molecules in the high-risk group. The relationship between the high circulating neutrophil to lymphocyte ratio and poor prognosis in lung cancer patients has been revealed in several studies (74–76). However, the plasticity of neutrophil function in the tumor microenvironment has not been fully uncovered and needs to be further investigated (77). In addition, LUAD patients in the high-risk group showed better sensitivity to chemotherapy, which may be associated with the increased proliferative and invasive capability in the ‘high-risk’ tumors. The above findings lead us to believe that the prognostic signature not only exhibits robustness and accuracy in clinical outcome prediction but also shows potential value in estimating the efficacy of chemotherapy and immunotherapy.

Some limitations of the present study should be mentioned. First, the establishment and validation of the prognostic signature were both based on retrospective analyses on public databases, which were biased. The robustness and accuracy of this study should be further confirmed in large prospective real-world studies. Besides, the correlation between some individual genes involved in the signature and lung adenocarcinoma remains unclear and warrants further exploration.

## Conclusion

In summary, we first developed and validated a novel signature incorporating both lipid metabolism-related and immune-related genes for LUAD patients. More importantly, the application of the prognostic model was extended to all stages of LUAD patients compared with previous studies. This is useful for predicting not only the prognosis but also the efficacy of chemotherapy and immune checkpoint inhibition.

## Data availability statement

The datasets presented in this study can be found in online repositories. The names of the repository/repositories and accession number(s) can be found in the article/**Supplementary Material**.

## Author contributions

YL, ZF, and JW designed the overall study. YW wrote the manuscript and performed all the experiments. JX performed the analysis of the high-throughput data. YF performed graphic processing and visualization and proofreading the manuscript. JG and FZ downloaded and pre-processed the data from the online databases. YT modified the language. RX and BZ assisted in data cleaning and analysis. YW, JX, and YF contributed equally to this study. All the listed authors directly, substantially, and intellectually contributed to the preparation of this manuscript and approved the publication.

## Funding

The research was funded by the National Natural Science Foundation of China (No. 81973795, 82174183); the Shanghai Municipal Natural Science Foundation (No. 19ZR1452200); the Shanghai Pujiang Program (No. 2020PJD057); and the YangFan project from the Science and Technology Commission of Shanghai Municipality (No. 22YF1445400).

## Acknowledgments

All authors would like to appreciate the TCGA and GEO databases for enabling the availability of high-quality data. We also acknowledge the American Journal Experts (AJE, www.aje.com) team for providing language help and writing assistance.

## Conflict of interest

The authors declare that the research was conducted in the absence of any commercial or financial relationships that could be construed as a potential conflict of interest.

## Publisher’s note

All claims expressed in this article are solely those of the authors and do not necessarily represent those of their affiliated organizations, or those of the publisher, the editors and the reviewers. Any product that may be evaluated in this article, or claim that may be made by its manufacturer, is not guaranteed or endorsed by the publisher.

## Supplementary material

Supplementary Figure 1 The optimal number of clusters in the TCGA cohort was determined by the *Silhouette* index.

Supplementary Figure 2 Validation of the unsupervised consensus clustering patterns in the external five GEO datasets.

Supplementary Figure 3 The consensus clustering algorithm distinguished LUAD patients in the high-risk and low-risk groups from healthy controls respectively.

Supplementary Figure 4 82 candidate genes with prognostic values were screened out by the Kaplan–Meier survival analysis.

Supplementary Figure 5 Validation of the prognostic signature model based on the GEO database. Comparison of the overall survival curves and time-dependent ROC curves in the GSE13213 dataset **(A)**, GSE31210 dataset **(B)**, GSE37745 dataset **(C)**, GSE68465 dataset **(D)**, and GSE72094 dataset **(E)**. **(F)** Meta-analysis of the prognostic value of the model based on the five GEO validation cohorts and LUAD training cohort.

Supplementary Figure 6 The tumor mutational burden score **(A)** and dysfunction score **(B)** between the high-risk and low-risk groups.

Supplementary Figure 7 Bar chart **(A)** and heatmap **(B)** displaying the difference in pathway activities enriched by GSVA between the high-risk and low-risk groups.

Supplementary Figure 8 Comparison of chemotherapeutic sensitivity between different risk groups. Estimated IC50 values of cisplatin **(A)**, docetaxel **(B)**, paclitaxel **(C)**, gemcitabine **(D)**, vinorelbine **(E)**, and bleomycin **(F)**.

Supplementary Table 1 Clinical characteristics of lung adenocarcinoma patients in the training datasets (TCGA) and the validation datasets (GEO).

Supplementary Table 2 List of lipid metabolism-related gene categories retrieved from the MSigDB database of GSEA (http://www.gsea-msigdb.org/gsea/msigdb/index.jsp).

Supplementary Table 3 List of specific immune-related and lipid metabolism-related genes included in the present study.

Supplementary Table 4 List of differentially expressed genes (DEGs) between lung adenocarcinoma and normal lung tissues based on the TCGA database.

Supplementary Table 5 List of 247 lipid metabolism-related DEGs by taking the intersection of DEGs of LUAD and lipid metabolism-related genes.
