# Supplementary figures and images for: Comprehensive analysis of a novel signature incorporating lipid metabolism and immune-related genes for assessing prognosis and immune landscape in lung adenocarcinoma
Source: Front Immunol. 2022 Aug 25;13:950001. doi: 10.3389/fimmu.2022.950001 (PMC9455632; doi:10.3389/fimmu.2022.950001)

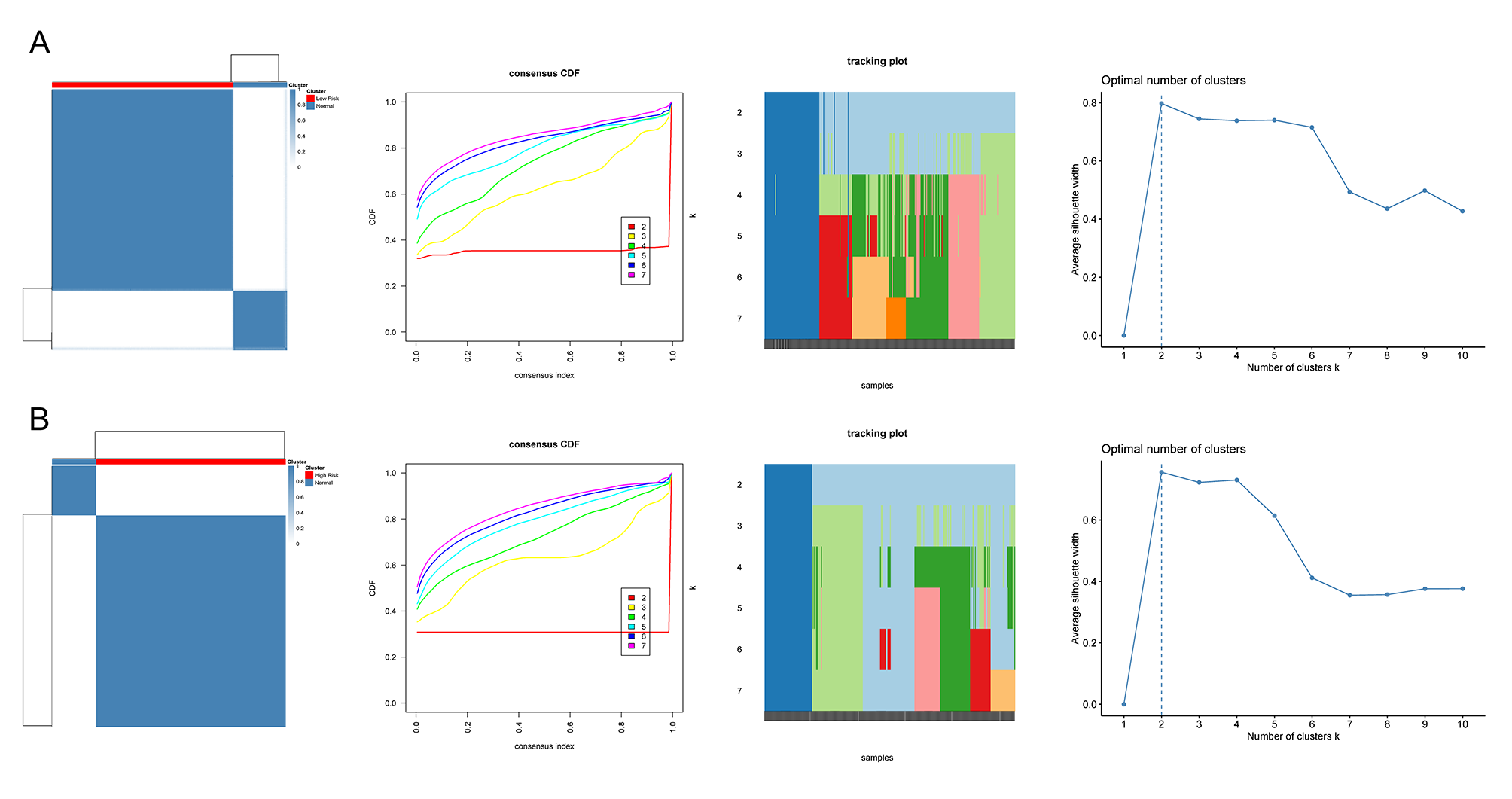

Supplement: Supplementary Figure 1 — The optimal number of clusters in the TCGA cohort was determined by the Silhouette index. [file Image_1.tif]

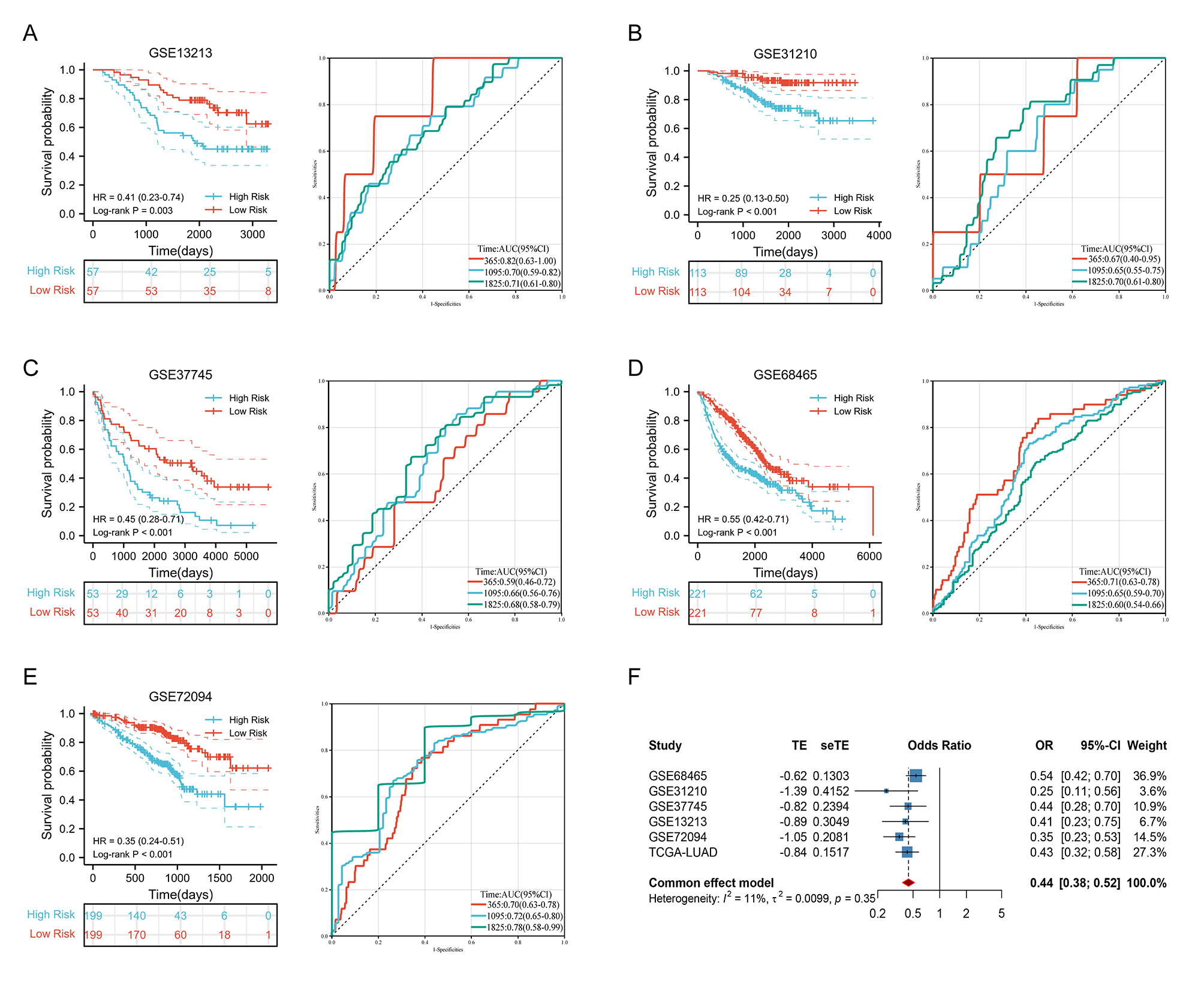

Supplement: Supplementary Figure 2 — Validation of the unsupervised consensus clustering patterns in the external five GEO datasets. [file Image_2.tif]

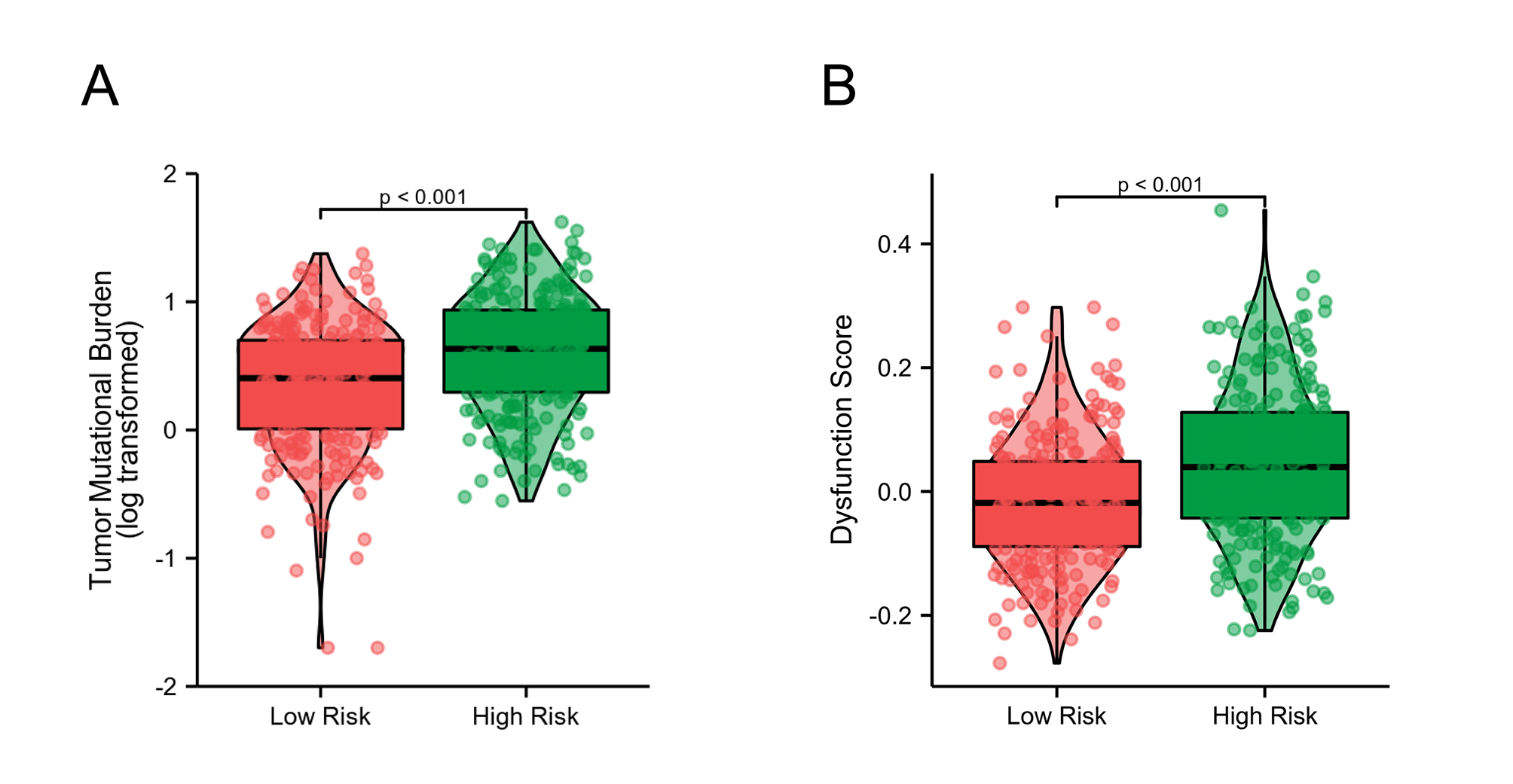

Supplement: Supplementary Figure 3 — The consensus clustering algorithm distinguished LUAD patients in the high-risk and low-risk groups from healthy controls respectively. [file Image_3.tif]

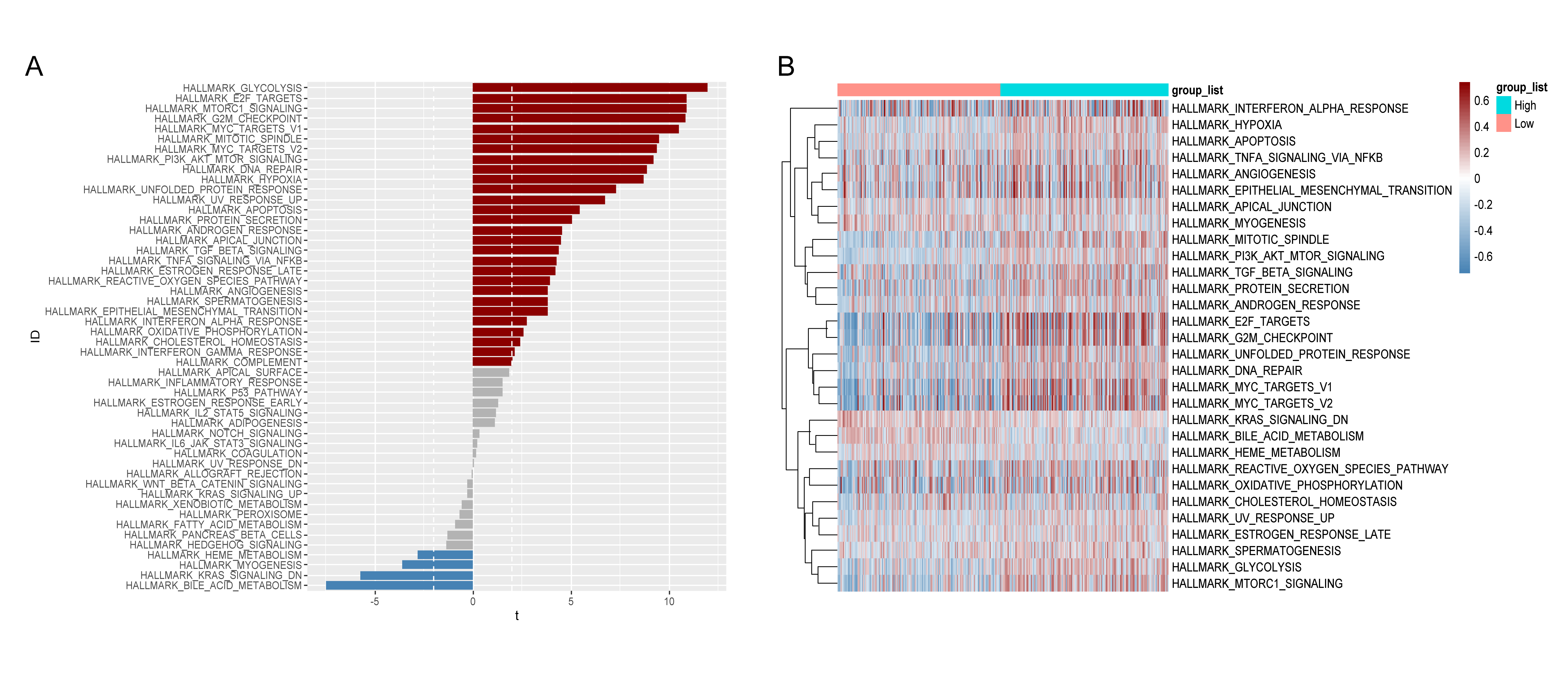

Supplement: Supplementary Figure 4 — 82 candidate genes with prognostic values were screened out by the Kaplan–Meier survival analysis. [file Image_4.tif]

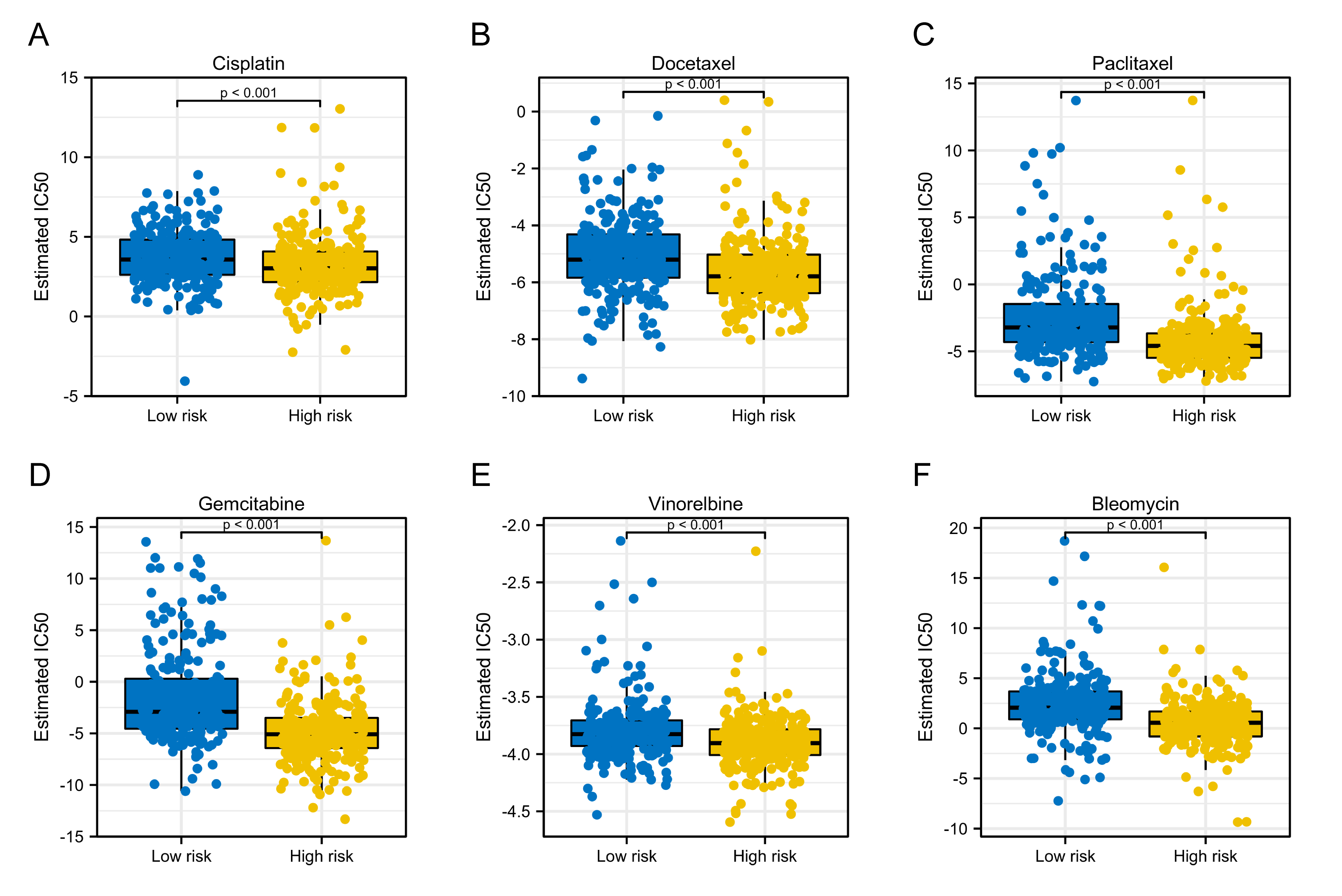

Supplement: Supplementary Figure 5 — Validation of the prognostic signature model based on the GEO database. Comparison of the overall survival curves and time-dependent ROC curves in the GSE13213 dataset (A), GSE31210 dataset (B), GSE37745 dataset (C), GSE68465 dataset (D), and GSE72094 dataset (E). (F) Meta-analysis of the prognostic value of the model based on the five GEO validation cohorts and LUAD training cohort. [file Image_5.tif]

Optimal number of clusters

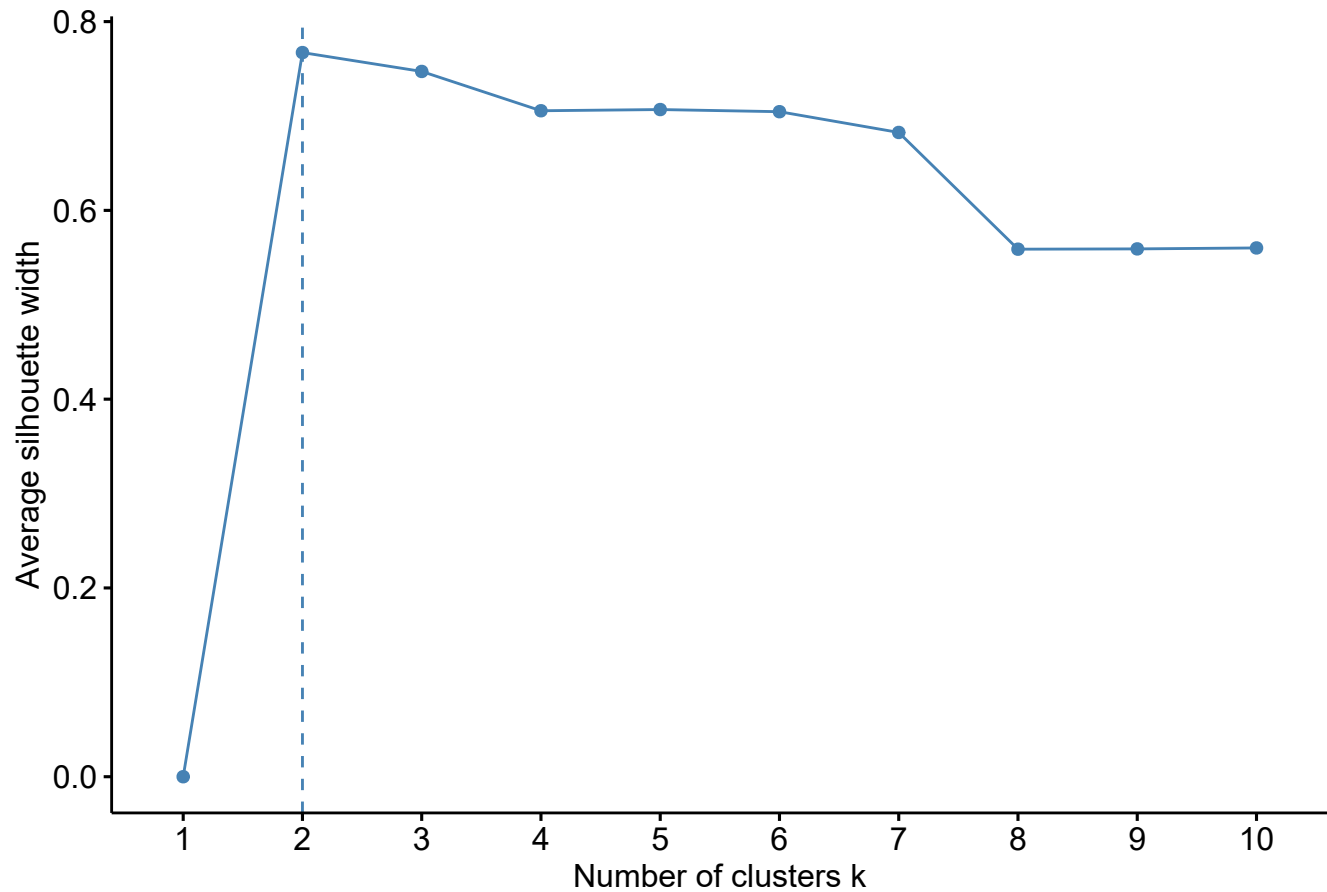

Supplement: Supplementary Figure 6 — The tumor mutational burden score (A) and dysfunction score (B) between the high-risk and low-risk groups. [file DataSheet_1.pdf]

A

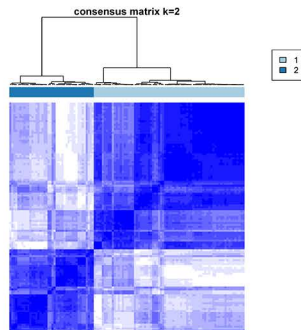

B

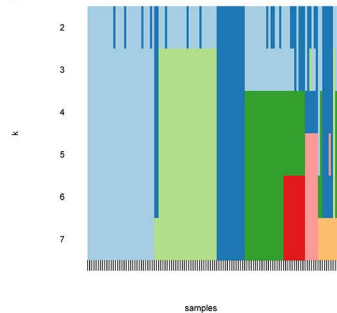

C

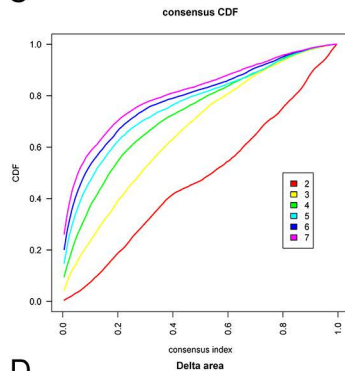

D

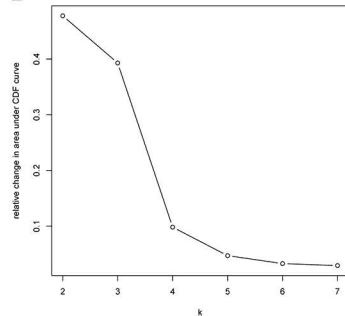

E

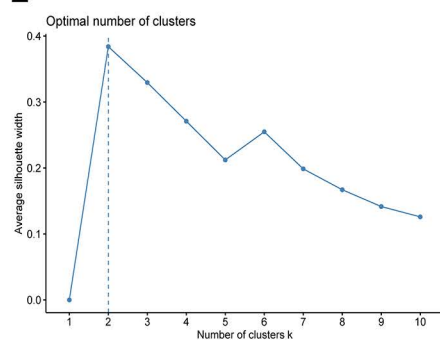

F

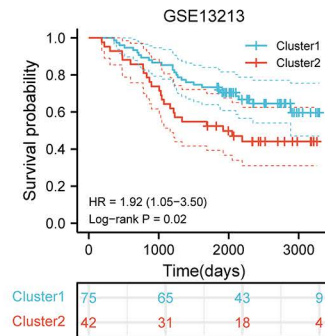

A

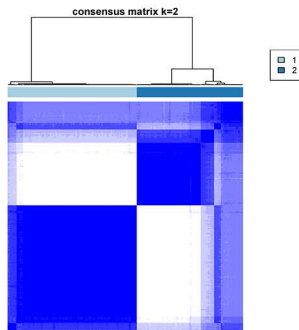

B

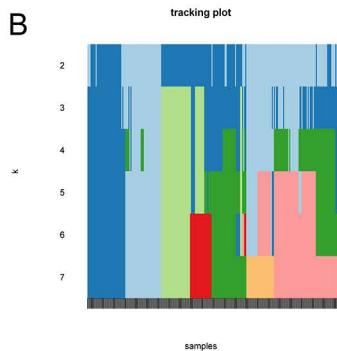

C

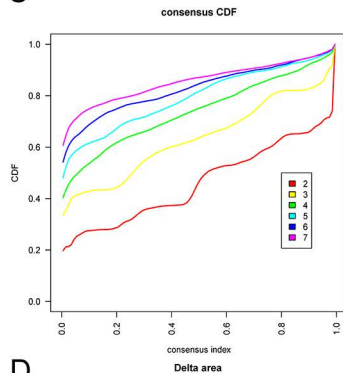

D

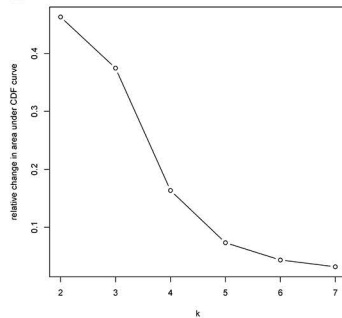

E

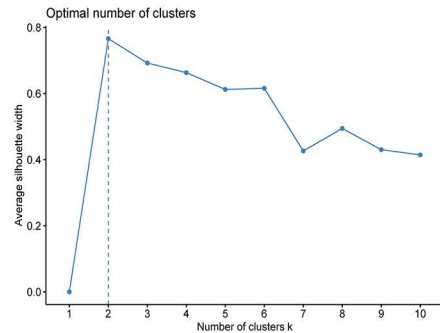

F

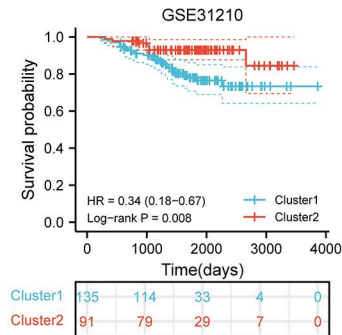

GSE31210

A

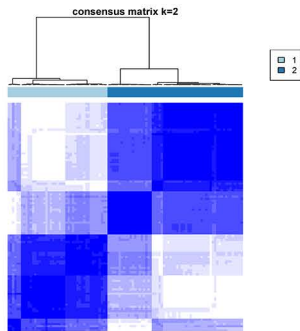

B

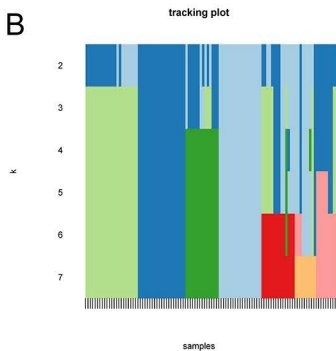

C

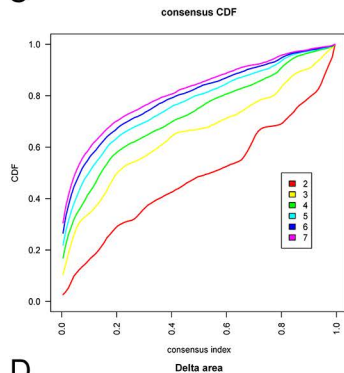

D

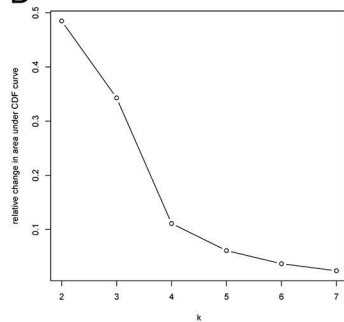

E

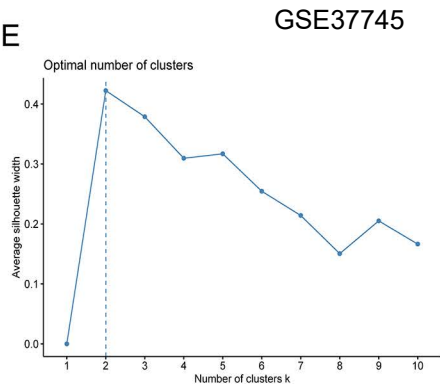

F

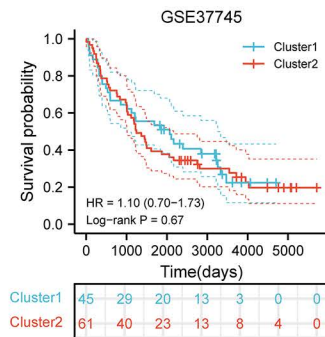

A

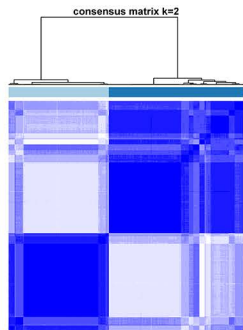

B

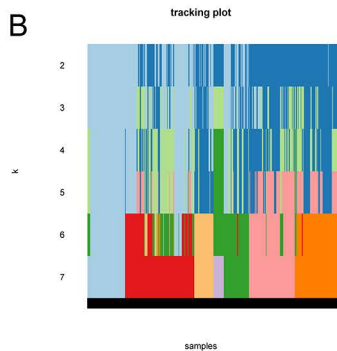

C

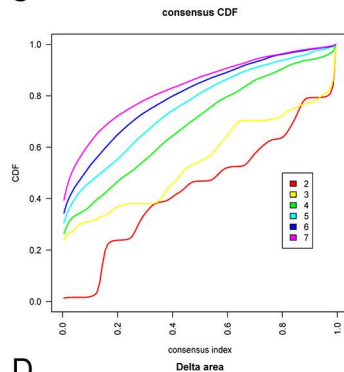

D

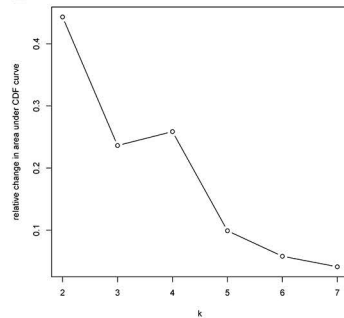

E

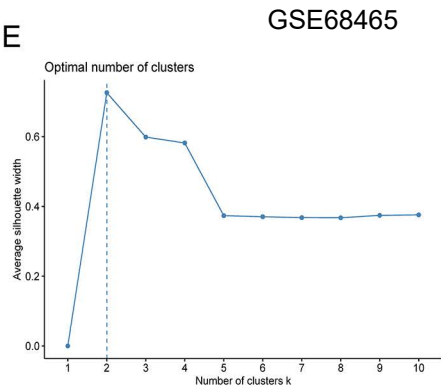

F

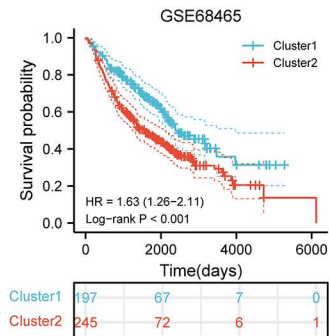

A

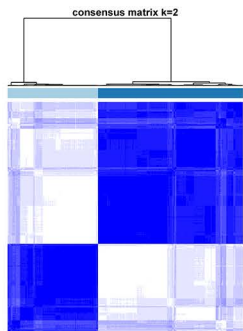

B

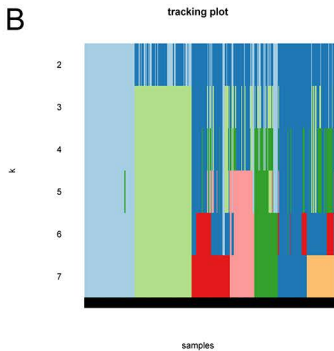

C

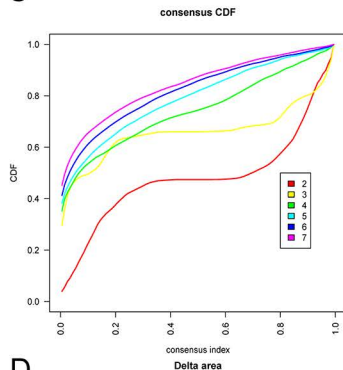

D

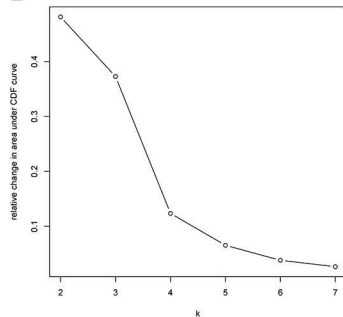

E

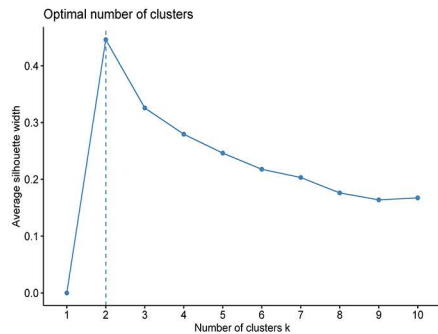

F

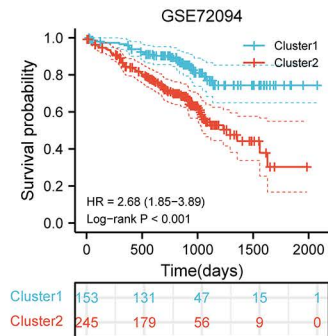

Supplement: Supplementary Figure 7 — Bar chart (A) and heatmap (B) displaying the difference in pathway activities enriched by GSVA between the high-risk and low-risk groups. [file DataSheet_2.pdf]

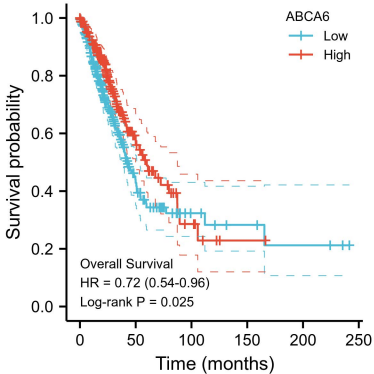

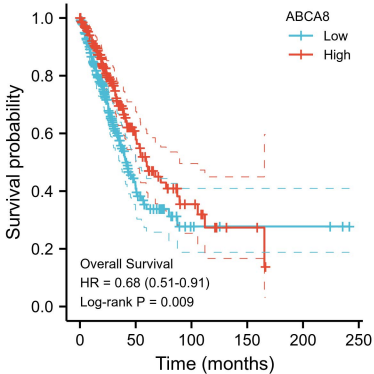

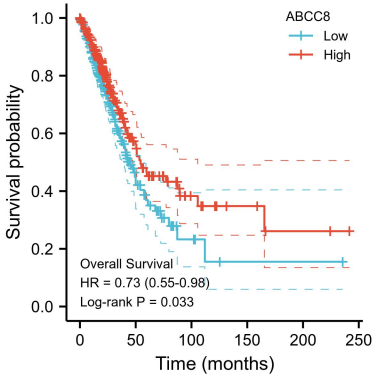

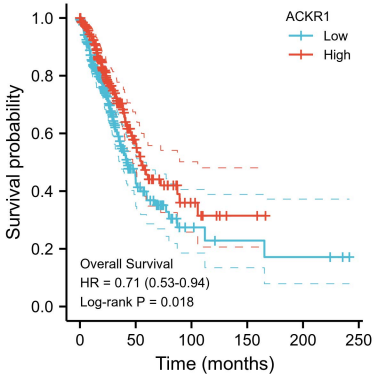

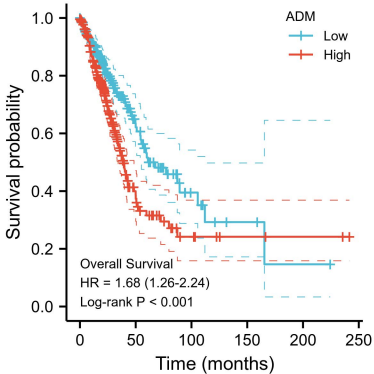

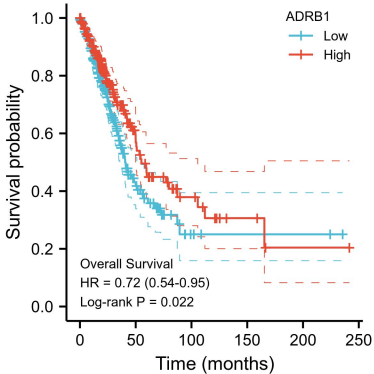

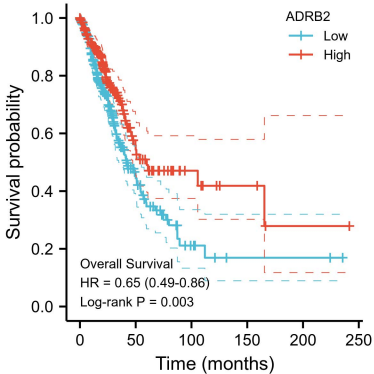

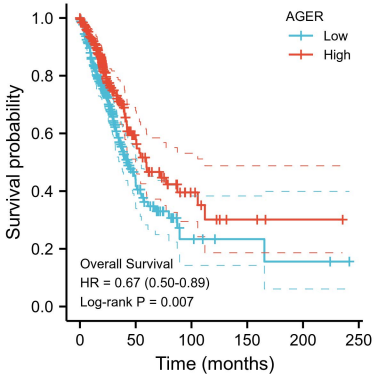

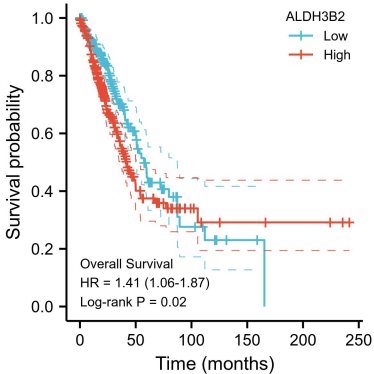

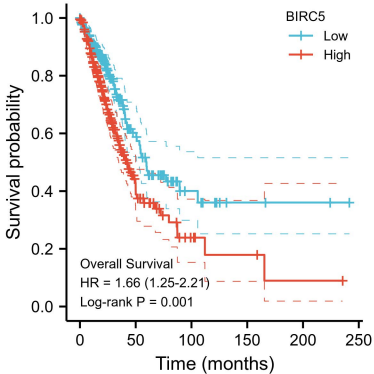

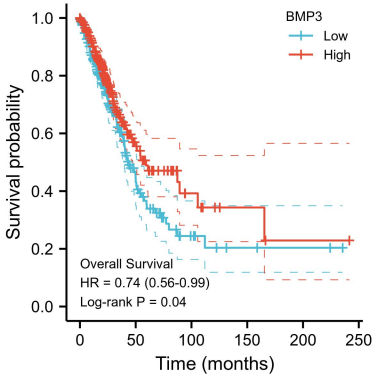

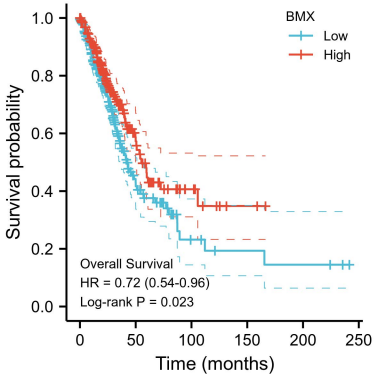

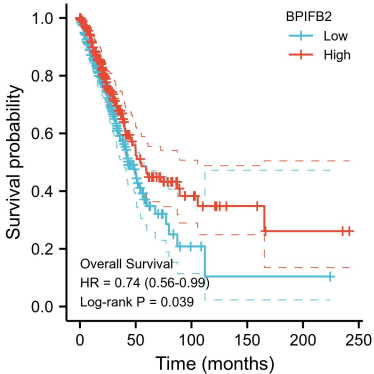

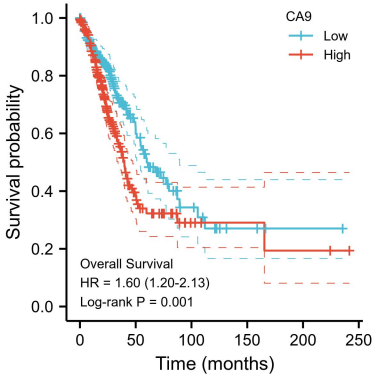

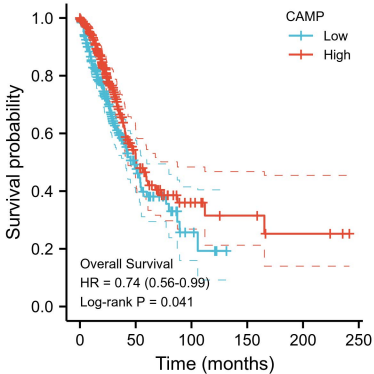

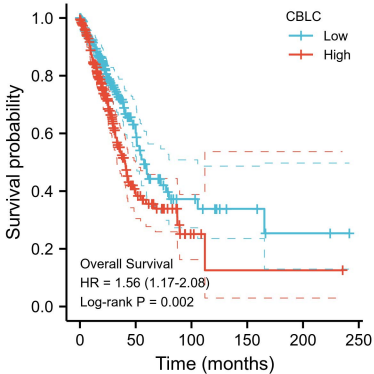

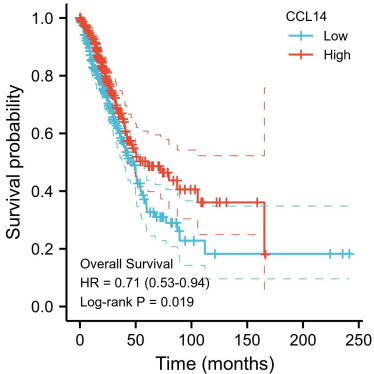

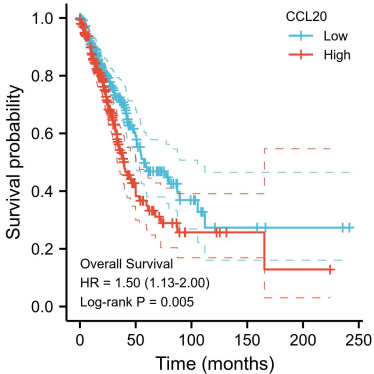

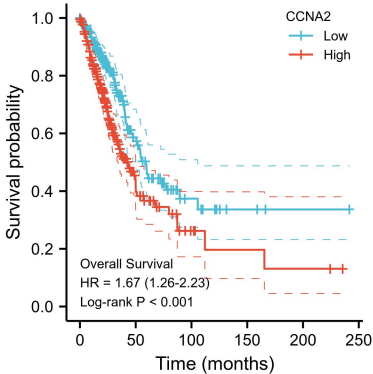

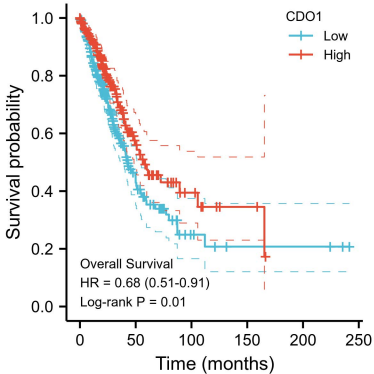

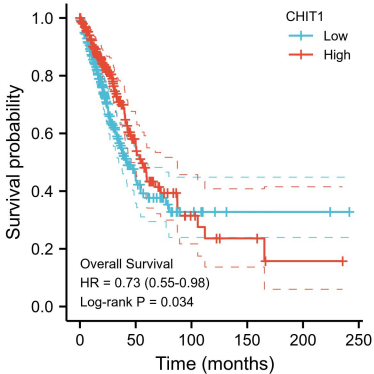

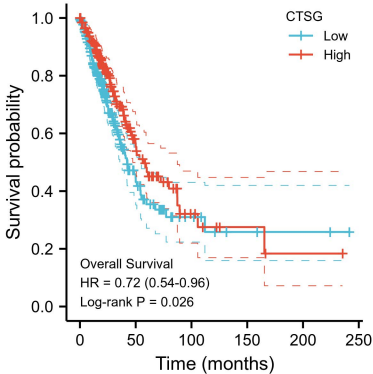

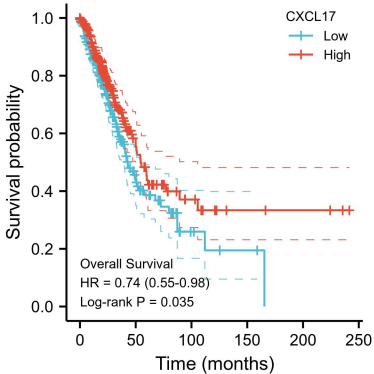

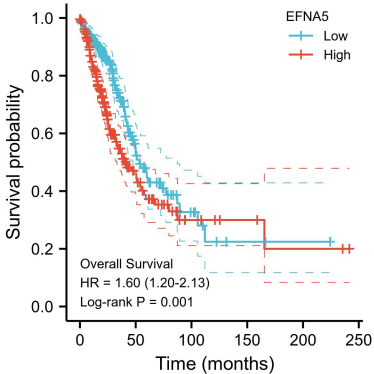

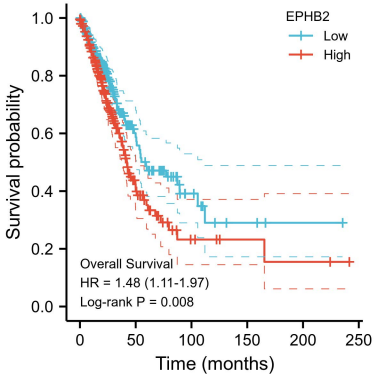

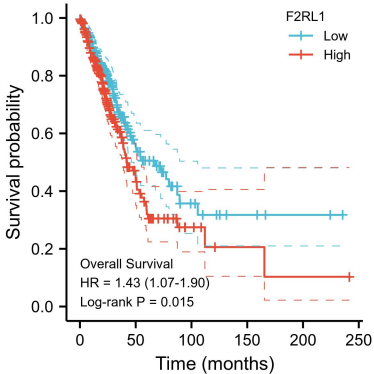

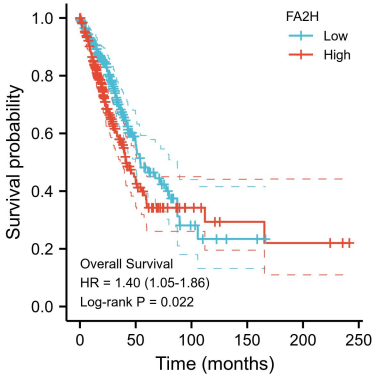

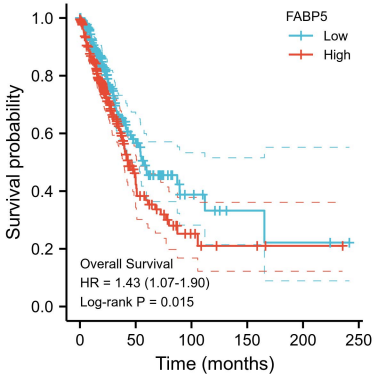

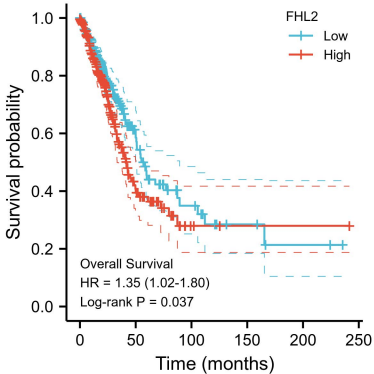

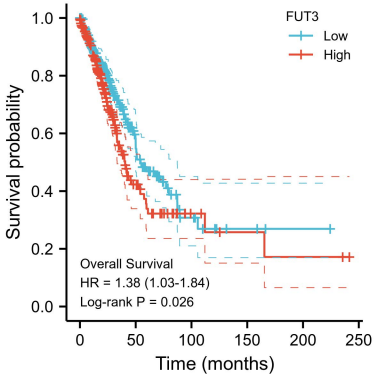

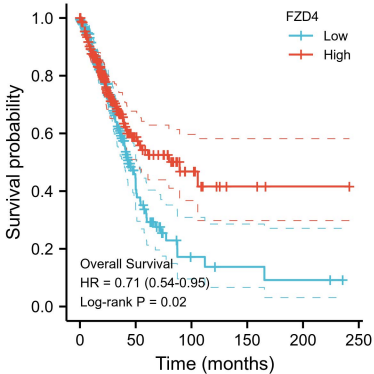

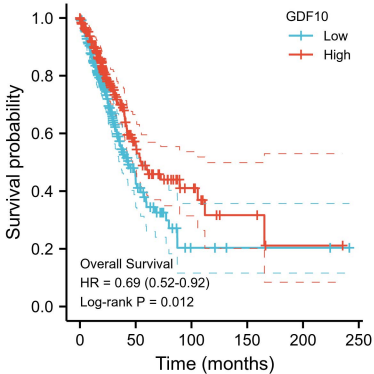

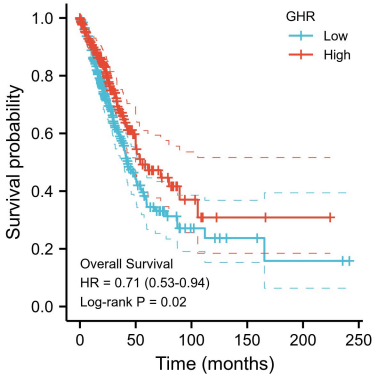

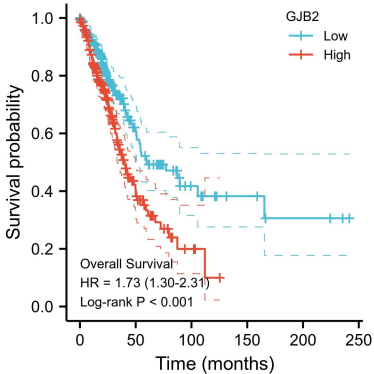

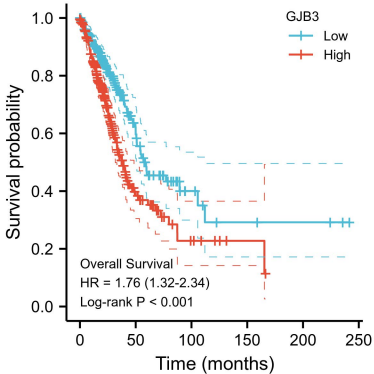

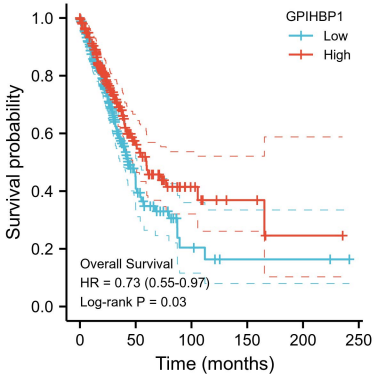

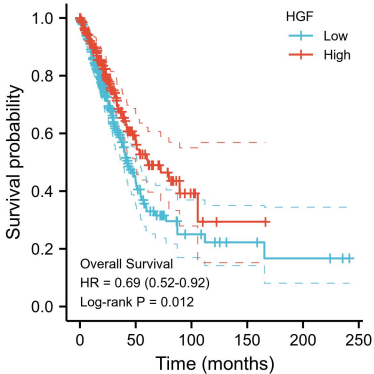

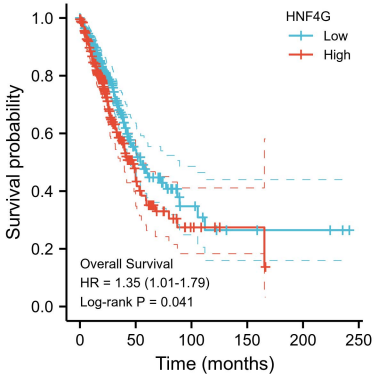

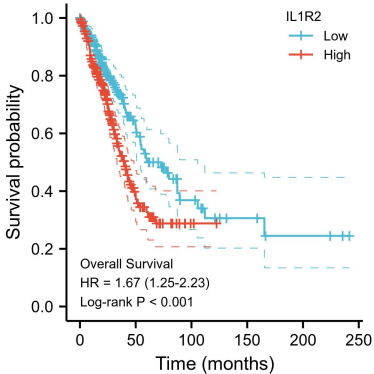

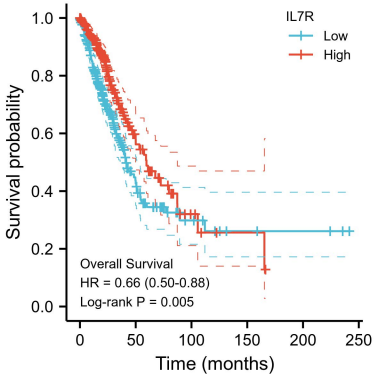

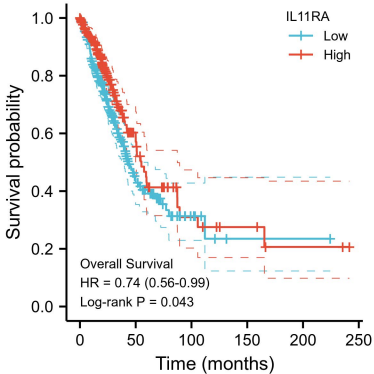

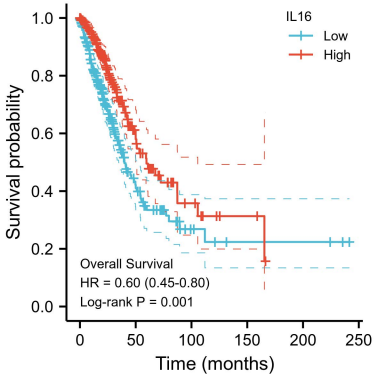

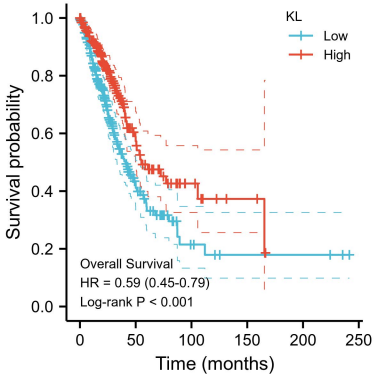

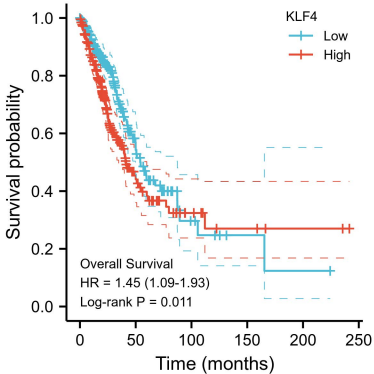

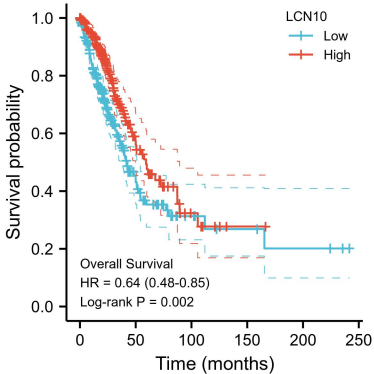

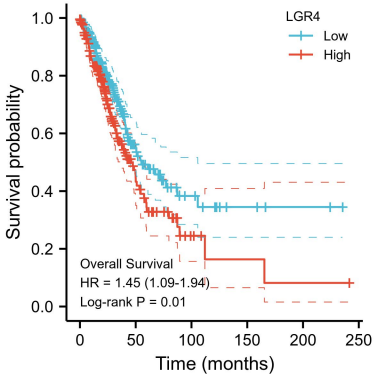

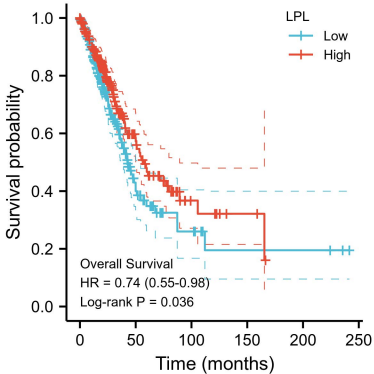

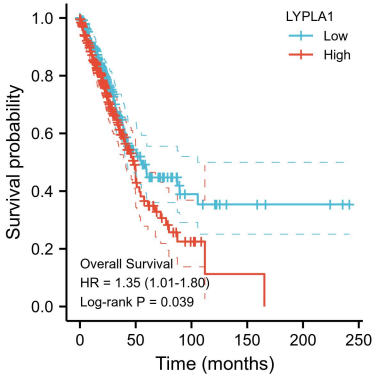

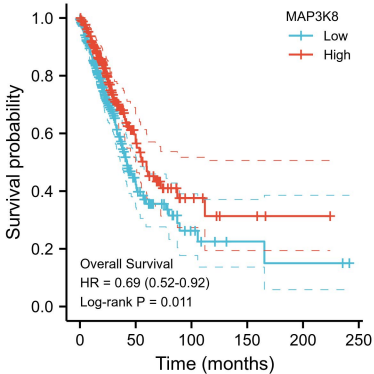

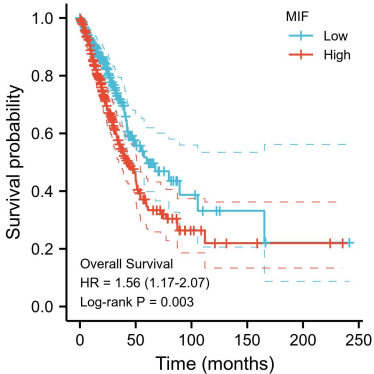

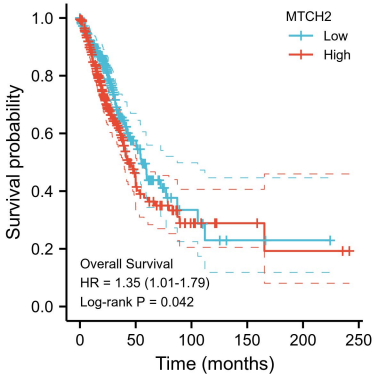

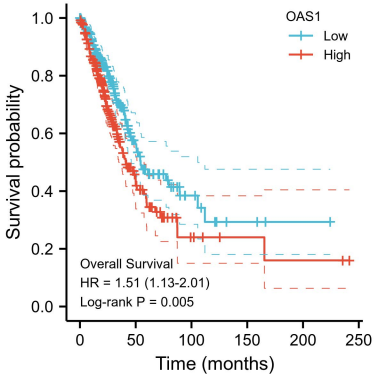

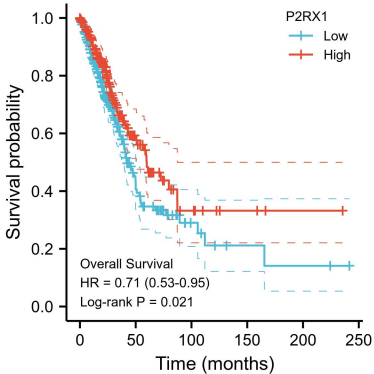

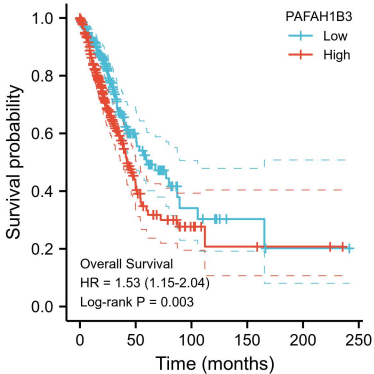

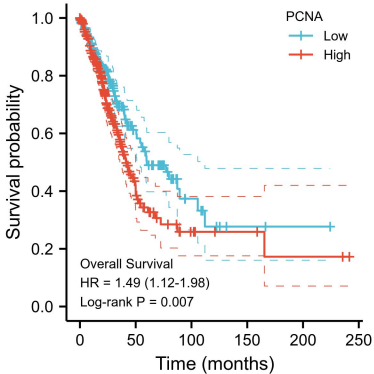

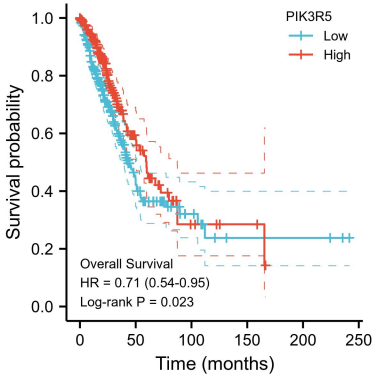

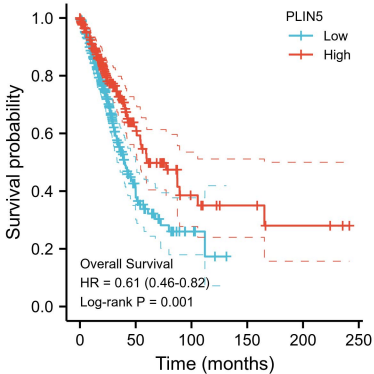

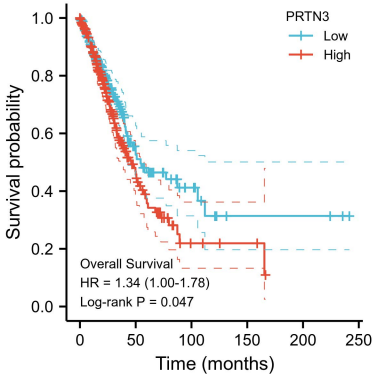

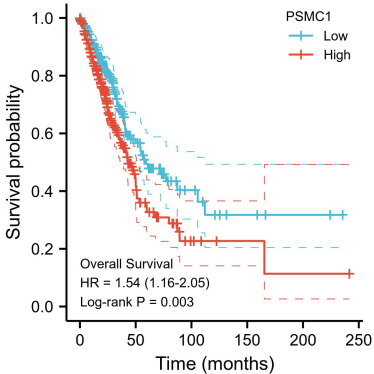

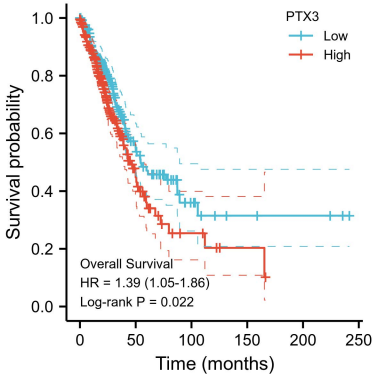

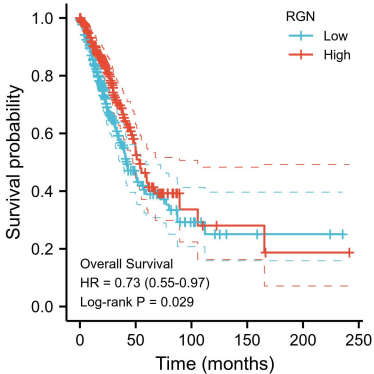

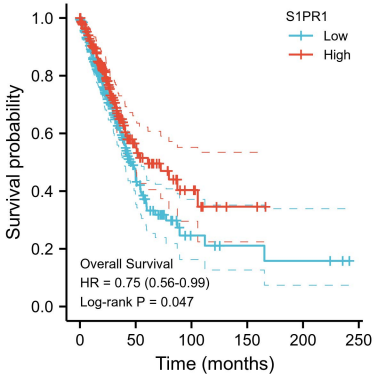

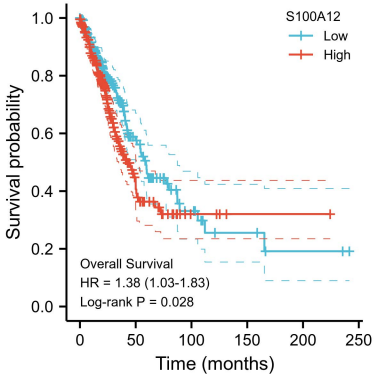

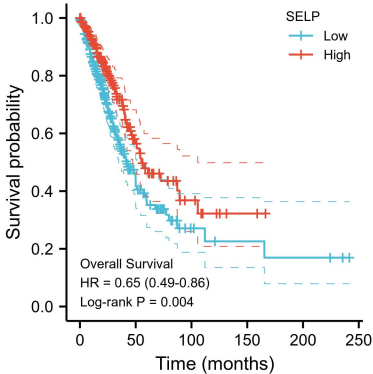

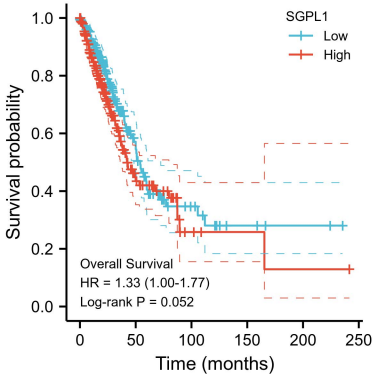

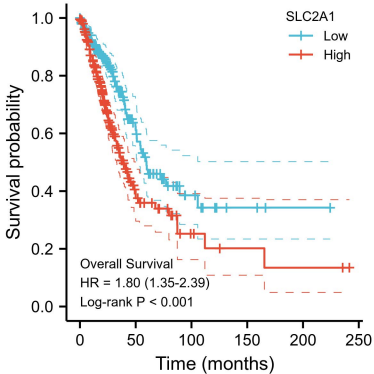

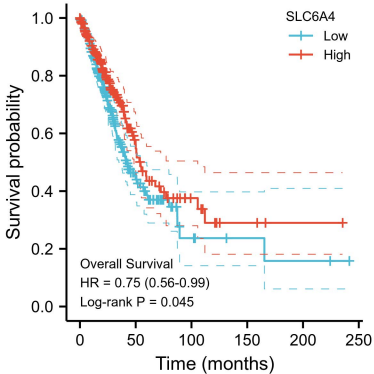

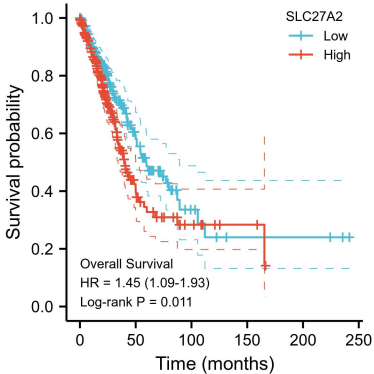

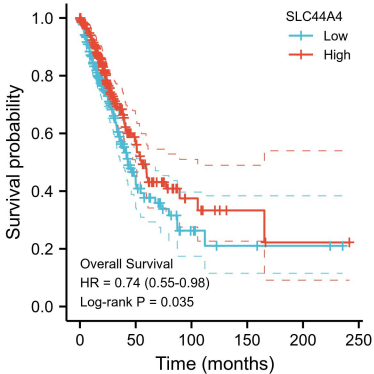

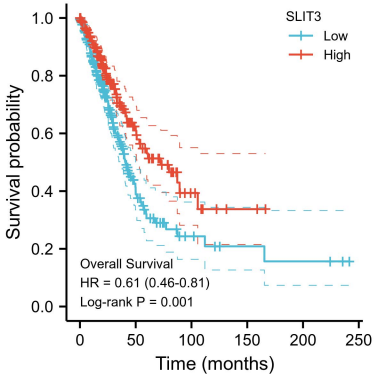

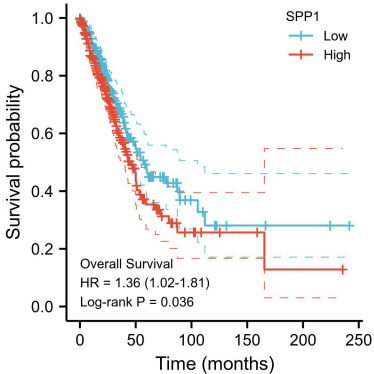

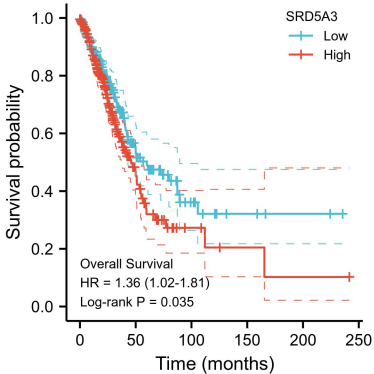

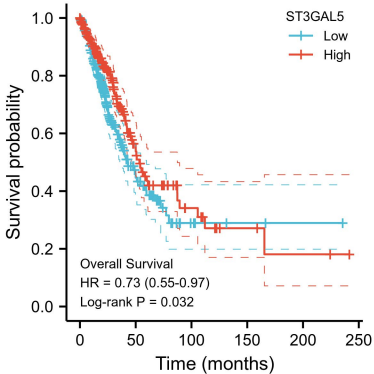

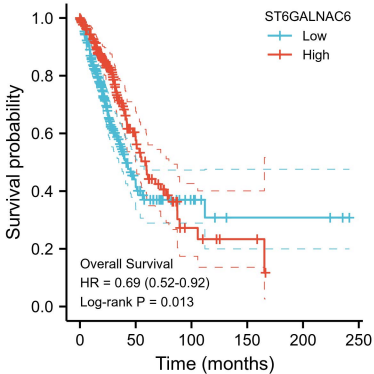

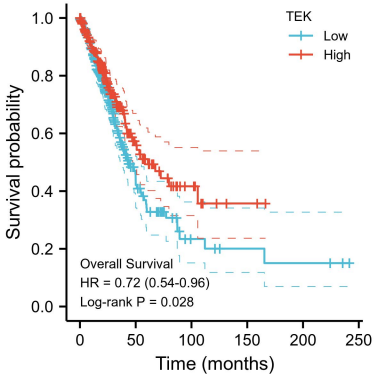

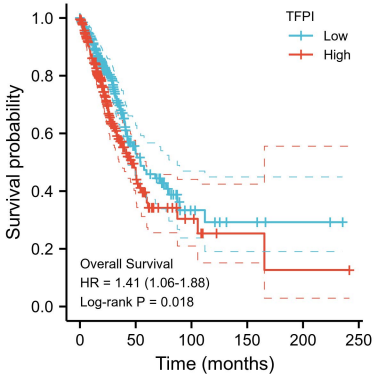

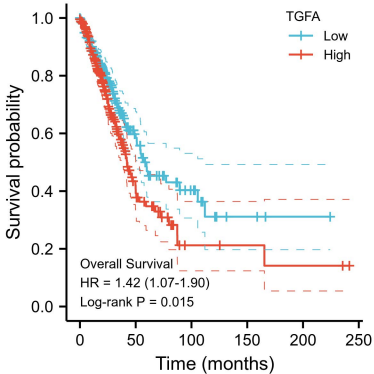

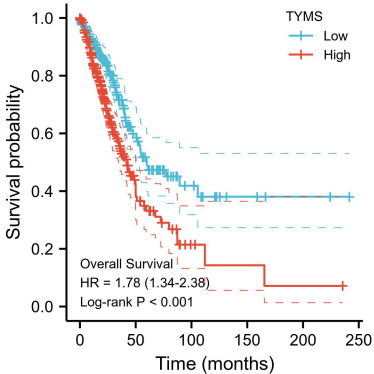

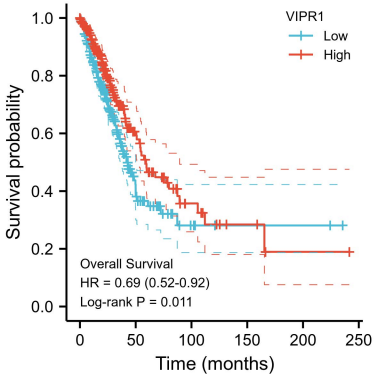

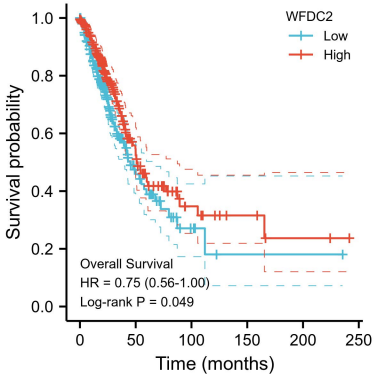

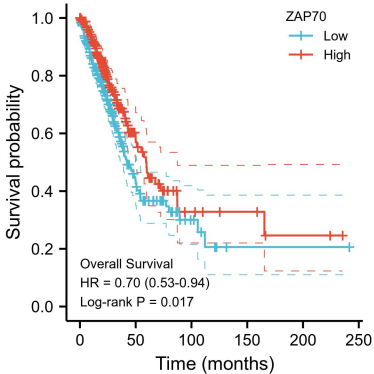

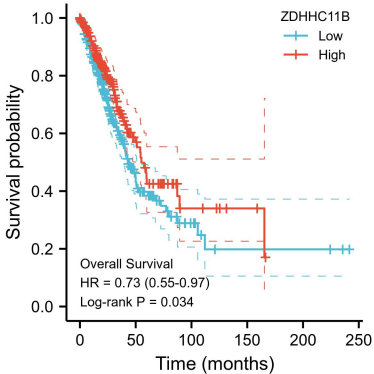

Supplement: Supplementary Figure 8 — Comparison of chemotherapeutic sensitivity between different risk groups. Estimated IC50 values of cisplatin (A), docetaxel (B), paclitaxel (C), gemcitabine (D), vinorelbine (E), and bleomycin (F). [file DataSheet_3.pdf]
