# Supplementary material for: Comprehensive analysis of a novel signature incorporating lipid metabolism and immune-related genes for assessing prognosis and immune landscape in lung adenocarcinoma
Source: Front Immunol. 2022 Aug 25;13:950001. doi: 10.3389/fimmu.2022.950001 (PMC9455632; doi:10.3389/fimmu.2022.950001)
Supplement: Supplementary Table 1 — Clinical characteristics of lung adenocarcinoma patients in the training datasets (TCGA) and the validation datasets (GEO). [file Table_1.docx]

**Supplementary Table1** Clinical characteristics of lung adenocarcinoma patients in the training datasets (TCGA) and the validation datasets (GEO)

| **Characteristics** | **TCGA**  **(*N* = 497)** | **GSE13213**  **(*N* = 114)** | **GSE31210**  **(*N* = 226)** | **GSE37745**  **(*N* = 106)** | **GSE68465**  **(*N* = 442)** | **GSE72094**  **(*N* = 398)** |
| --- | --- | --- | --- | --- | --- | --- |
| **Age** |  |  |  |  |  |  |
| <65 | 214 | 74 | 176 | 54 | 213 | 107 |
| >=65 | 263 | 40 | 50 | 52 | 229 | 291 |
| **Gender** |  |  |  |  |  |  |
| Male | 228 | 59 | 153 | 46 | 222 | 176 |
| Female | 269 | 55 | 181 | 60 | 219 | 222 |
| **Stage** |  |  |  |  |  |  |
| I | 267 | 78 | 168 | 70 | 275 | 254 |
| II | 123 | 13 | 58 | 19 | 95 | 67 |
| III | 81 | 23 | / | 13 | 68 | 57 |
| IV | 25 | / | / | 4 | / | 15 |
| Unknown | 1 | / | / | / | 4 | 5 |
| **Status** |  |  |  |  |  |  |
| Alive | 317 | 67 | 191 | 29 | 206 | 285 |
| Dead | 180 | 47 | 35 | 77 | 236 | 113 |
| **Relapse** |  |  |  |  |  |  |
| Yes | 24 | 56 | 64 | 26 | / | / |
| No | 472 | 57 | 162 | 27 | / | / |
| Unknown | 1 | 1 | / | 53 | / |  |
| **P53** |  |  |  |  |  |  |
| Wild Type | 240 | 77 | / | / | / | 301 |
| Mutation | 257 | 36 | / | / | / | 97 |
| Unknown | / | 1 | / | / | / | / |
| **EGFR** |  |  |  |  |  |  |
| Wild Type | 427 | 70 | 99 | / | / | 357 |
| Mutation | 70 | 44 | 127 | / | / | 41 |
| **KRAS** |  |  |  |  |  |  |
| Wild Type | 355 | 100 | 206 | / | / | 259 |
| Mutation | 142 | 14 | 20 | / | / | 139 |
